# Unravel the molecular basis underlying inflorescence color variation in *Macadamia* based on widely targeted metabolomics

**DOI:** 10.3389/fpls.2025.1533187

**Published:** 2025-03-25

**Authors:** Lidan Gong, Haiqing Zhang, Jing Ma, Zhiqiang Li, Tingyu Li, Chao Wu, Yang Li, Liang Tao

**Affiliations:** Yunnan Institute of Tropical Crops, Jinghong, China

**Keywords:** *Macadamia integrifolia*, inflorescences color, widely targeted metabolomics, WGCNA, flavonoids

## Abstract

*Macadamia integrifolia*, a perennial evergreen crop valued for its nutritious nuts, also exhibits a diverse range of inflorescence colors that possess both ornamental and biological significance. Despite the economic importance of macadamia, the molecular mechanisms regulating flower coloration remain understudied. This study employed a combination of metabolomic and biochemical approaches to analyze metabolites present in inflorescences from 11 Macadamia cultivars, representing distinct color phenotypes. A total of 787 metabolites were identified through the use of ultra-high-performance liquid chromatography–tandem mass spectrometry (UPLC–MS/MS), the majority of which were phenolic acids, flavonoids, and flavonols. Principal component analysis and clustering yielded a classification of the samples into three major flower color groups. The differential metabolites were found to be enriched in pathways such as flavonoid, flavonol, and phenylpropanoid biosynthesis, which have been demonstrated to be key contributors to color variation. Moreover, weighted gene co-expression network analysis (WGCNA) identified metabolite modules that were strongly associated with specific flower colors. This revealed that key compounds, including kaempferol, quercetin derivatives, and anthocyanins, were the primary drivers of pigmentation. This study provides a comprehensive framework for understanding the genetic, biochemical, and environmental factors influencing macadamia flower color. These findings contribute to the theoretical understanding of macadamia reproductive biology and have practical implications for molecular breeding, ornamental enhancement, and optimizing pollinator attraction to improve crop yield and ecological sustainability.

## Introduction

The macadamia tree (*Macadamia integrifolia*) is indigenous to Australia and is classified within the family Proteaceae. The macadamia nut is renowned for its edible properties and is considered a valuable source of nutrition due to its high levels of unsaturated fatty acids. This has resulted in the macadamia being designated as the “king of nuts” (Hu et al., 2019). In response to the growing global demand for healthy diets and high-quality foods, macadamia nuts have emerged as a highly sought-after economic crop. Their applications extend beyond the food industry, with increasing use in the pharmaceutical and cosmetic sectors, indicating a broad market potential (Ako et al., 1995). In recent years, the macadamia industry in China has undergone a period of rapid expansion, particularly in the Yunnan and Guangxi provinces, where the planted area has exceeded 300,000 hectares, becoming a significant contributor to agricultural economic growth (Tu et al., 2021). Globally, high-yielding cultivars such as ‘Beaumont’, ‘A4’, and ‘HAES 816’ dominate commercial production, with inflorescence colors ranging from white to pale pink. For instance, ‘HAES 816,’ widely cultivated in Australia and South Africa, produces light-yellow inflorescences that correlate with enhanced pollinator attraction and nut yield (Trueman et al., 2022; Langdon et al., 2020). These cultivars exemplify the interplay between floral pigmentation and agricultural productivity, underscoring the need to decode the metabolic basis of color variation for breeding optimization. During the growth and development of macadamia, the flowers serve a pivotal function as reproductive organs, exerting a considerable influence on the yield and quality of the nuts. Prior research on macadamia has predominantly concentrated on the enhancement of yield and quality, with comparatively limited attention directed towards the flowers themselves. This has resulted in the underutilization of their inherent value. The flowers of macadamia are distinguished by elongated racemes adorned with small flowers in white, pink, or purple, conferring considerable ornamental value and establishing them as significant ornamental trees. Additionally, macadamia flowers contain a variety of compounds, including flavonoids, phenolic substances, polysaccharides, saponins, proteins, and amino acids. It has been demonstrated that phenolic compounds present in the flowers exhibit antioxidant properties, which assist in the neutralization of free radicals and may contribute to a reduction in the risk of chronic diseases (Shahidi, 2003; Ali et al., 2021). Furthermore, flavonoids, which have been extensively investigated for their anti–inflammatory and anticancer properties (Dai et al., 2017), contribute to the medicinal potential of macadamia flowers. The essential oils and other small molecular compounds that can be extracted from macadamia flowers are also of value for use in cosmetics and functional foods, thereby enhancing their economic worth (Castada et al., 2020).

In the field of plant reproductive biology, flower color is regarded as a pivotal factor influencing reproductive success (Borghi et al., 2017b). Color serves not only as an attractant for specific pollinators but is also closely related to the adaptability and competitive ability of plants (Liu et al., 2018). Consequently, an investigation into the underlying mechanisms of flower color formation holds significant theoretical and practical importance. For example, the manipulation of pigment metabolic pathways to alter flower color can enhance the aesthetic appeal, market value, and attractiveness to specific pollinators of the plant in question (Borghi and Fernie, 2017a). The process of flower color formation is a complex process that involves the synthesis of pigments, genetic regulation, and environmental influences (Ballester et al., 2010; Albert et al., 2011). Research indicates that pigments such as anthocyanins, flavonoids, and carotenoids play a pivotal role in determining the color of plant inflorescences. The accumulation and distribution of these pigments are subject to strict regulation by a number of biochemical pathways. It has been demonstrated that the anthocyanin biosynthetic pathway is regulated by enzymes such as phenylalanine ammonia–lyase (*PAL*), chalcone synthase (*CHS*), dihydroflavonol reductase (*DFR*), and anthocyanin synthase (*ANS*), which permit flowers to exhibit a wide range of colors based on the regulation of these key genes (Tanaka et al., 2008). Furthermore, the specific types and concentrations of anthocyanins can vary depending on the plant species and environmental factors. It has been demonstrated that environmental factors such as light exposure, temperature, and soil pH can exert a considerable influence on pigment synthesis and accumulation (Jiang et al., 2016; Liang et al., 2020). For example, under conditions of ample light, certain plants produce greater quantities of anthocyanins, resulting in more vibrant colors. Conversely, low temperatures may increase flavonoid accumulation, shifting color towards yellow–green (Løvdal et al., 2010). These findings elucidate the biochemical basis of pigment synthesis and also demonstrate how plants adapt to environmental changes.

The advent of high–throughput omics technologies has significantly accelerated research on the genetic basis of flower color. Genomic studies have identified candidate genes that are involved in the formation of flower color through the differential analysis of gene sequences and expressions. These include transcription factors such as APETALA2 (AP2), MYB, and bHLH, which regulate pigment synthesis (Li et al., 2019; Lai et al., 2016). Metabolomic techniques, including mass spectrometry and liquid chromatography, are employed to identify and quantify metabolites in flowers, thereby providing data support for elucidating pigment synthesis pathways and metabolic networks (Feng et al., 2024; Sun et al., 2023). These research methodologies not only enhance the accuracy and resolution of studies on flower color but also open new avenues for the analysis of different pigment synthesis pathways. At present, research on the inflorescence color of macadamia is still in its early stages. Prior research has demonstrated that macadamia flowers predominantly display hues of white, light yellow, and pink, with variations in hue and shade across different varieties. However, the specific pigments involved, the underlying metabolic pathways, and the genetic regulatory mechanisms remain unclear (Wang et al., 2023). Moreover, research on macadamia inflorescences lacks a comprehensive and systematic analysis of pigment content in relation to environmental variables. Consequently, some studies have commenced an investigation into the distribution of pigments in macadamia flowers and their potential biological significance, particularly in attracting pollinators and enhancing reproductive success (Trueman et al., 2022).

Ultra–high–performance liquid chromatography–tandem mass spectrometry (UPLC–MS/MS), renowned for its exceptional detection precision and robust isomer identification capabilities, has been extensively utilized to elucidate the biosynthetic pathways of metabolites in flowers of diverse colors (Qiu et al., 2023; Zeng et al., 2024). In this study, we selected 11 macadamia nut cultivars with different inflorescence colors and employed a comprehensive approach to identify the metabolites present in these inflorescence samples. To elucidate the differences in secondary metabolite distributions in inflorescences of different colors, we utilized a range of data analysis methods. Finally, we explored the potential molecular mechanisms underlying the formation of different flower colors. The results of this study offer new insights into the major metabolites that influence macadamia inflorescence color. These findings provide a valuable reference for future multifaceted applications and molecular breeding of macadamia flowers.

## Materials and methods

### Plant materials

**Figure 1:**
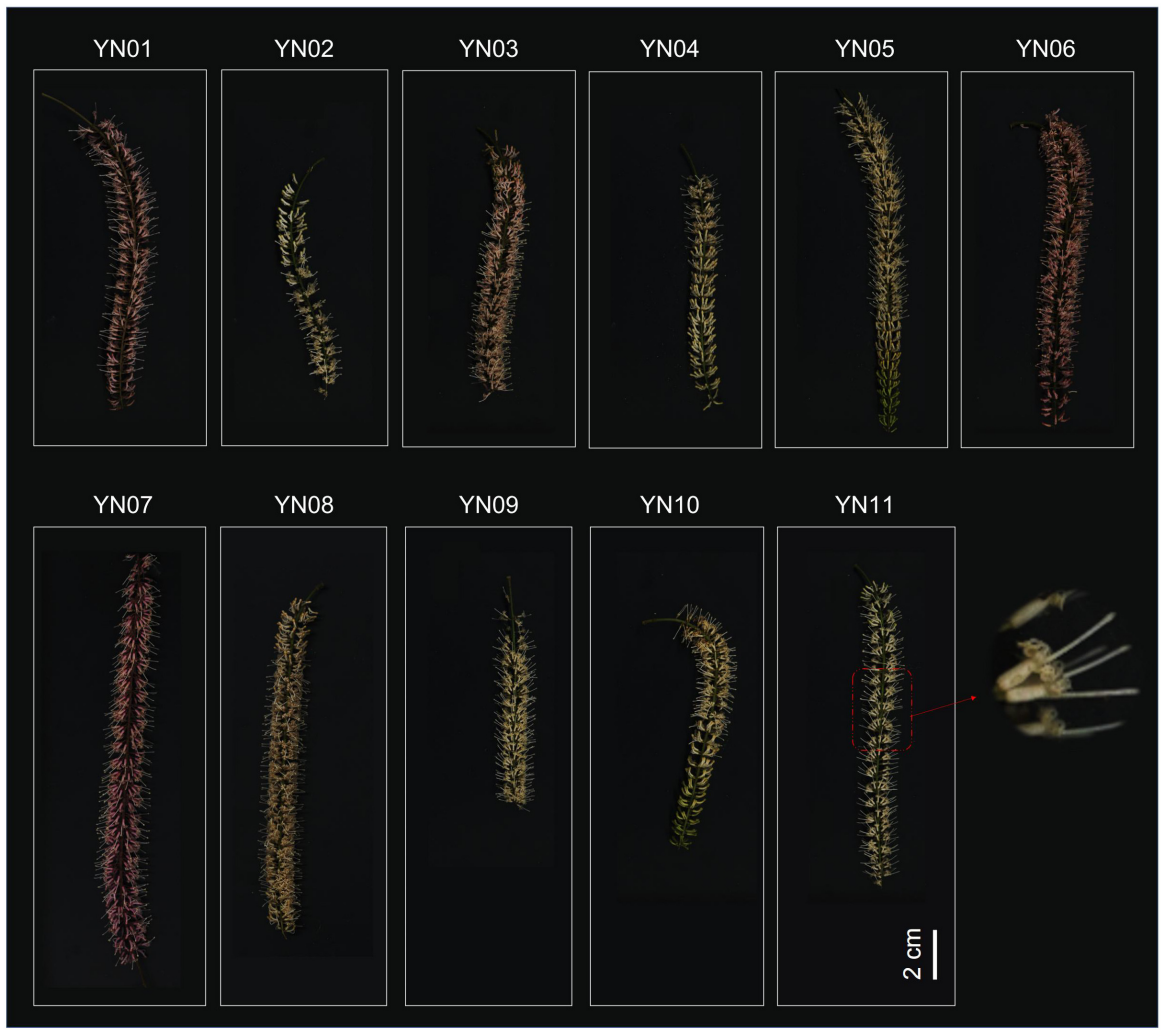
Phenotypic characterization of inflorescences of 11 different macadamia varieties (YN01-YN11).

The inflorescences of Macadamia that were utilized in this study were procured in February 2023 from the Macadamia germplasm repository at the Tropical Crop Science Research Institute in Yunnan Province (longitude 100°46′42.24″, latitude 22°00′50.4″). The mean annual temperature in Jinghong, Xishuangbanna Dai Autonomous Prefecture, is between 18°C and 22°C, with an average annual precipitation of between 1136 and 1513 millimeters. The region is notable for its richness in macadamia germplasm resources. Inflorescences exhibiting a range of floral colors were procured from 11 distinct Macadamia cultivars, each labeled sequentially as YN01 to YN11 (**Figure 1**). For each cultivar, we collected three biological replicates. The choice of three biological replicates was based on common practices in plant metabolomics research (Wang et al., 2023; Xiao et al., 2023). Three replicates are often considered a balance between experimental workload and statistical power in similar studies. This number has been used in previous research to effectively capture biological variation within and between samples in plant metabolomic analyses. For instance, Wang et al. investigated the metabolome of macadamia flower colour, employing three biological replicates in each treatment group to identify significant metabolic differences. In a similar manner, Xiao et al. utilised three replicates to analyse metabolite changes in sweetpotato of different colours in their study of sweetpotato. These cultivars exhibit a wide range of phenotypic variations in flower color, providing a rich source of samples for an in–depth analysis of the diversity in Macadamia inflorescence color. All samples were whole flowers, immediately frozen in liquid nitrogen, and stored at –80°C for future use. Each cultivar was subjected to three biological replicates for the measurement of total phenols, flavonoids, anthocyanins, and tannin content. Additionally, identical samples were used for comprehensive targeted metabolomic analyses. Each replicate included at least six flowers collected from two or three different Macadamia trees, mixed in equal proportions.

### Measurement of total phenols, flavonoids, anthocyanins, and tannin content

The total phenol content in the macadamia inflorescences was determined using the Folin–Ciocalteu method (Xu et al., 2008). Briefly, the macadamia nut flower samples to be tested were placed in a mortar and pestle, ground into powder with liquid nitrogen, accurately weighed 1.0g into a centrifuge tube, 10mL of 70% acetone solution was added, mixed and then extracted in a 37°C water bath for 1h, followed by centrifugation for 20min at 4°C and 2200r, and then finally the supernatant was filtered and used for the determination of the total phenol content. The results are expressed as milligrams of gallic acid equivalents (GAE) per gram of dry sample. The total flavonoid content was determined using the NaNO_2_–Al(NO_3_)_3_ colorimetric method (Meda et al., 2005). Similar to the determination of total phenol content, the sample to be tested was ground, 1.0g was accurately weighed into a centrifuge tube, 10mL of 70% acetone solution was added, and the extract was immersed in a 37°C water bath for 1h, then centrifuged for 20min at 4°C and 2200r, and the supernatant was filtered for the determination of total flavonoids content. The mean of three readings was employed for analysis, with the results expressed as milligrams of rutin equivalents (RE) per gram of dry weight. The anthocyanin content was quantified in accordance with a previously established protocol (Meng et al., 2001), with the absorbance measured at 530 nm using a spectrophotometer. Take 10g of macadamia inflorescences, grind them into powder after quick freezing with liquid nitrogen, add 10mL 0.1mol-L hydrochloric acid - methanol solution (methanol to hydrochloric acid ratio 1:3) to dissolve, mix well and ultrasonicate for 60min (25kHz). Then add 8mL distilled water, mix and centrifuge (12000r/min, 5min), transfer the supernatant into a centrifuge tube, add an equal volume of chloroform, mix upside down, stand and centrifuge again (12000r/min, 5min), separate the aqueous and chloroform phases, and collect the aqueous phase solution as the extract for the determination of anthocyanin content. This assessment was conducted relative to a blank control. The total tannin content (TTC) was determined using the Bate–Smith method, as previously described (Donkor et al., 2019). Accurately weigh 1.0g of the powder of the inflorescence sample into a 100mL triangular flask, add 50mL of 75% dimethylformamide solution, sealed with a stopper and shaken on a shaker for 60min, then filtered through filter paper and used for the determination of tannin content. The TTC is expressed as the mass of tannins per mass of dry matter (mg g^–1^). All analyses were conducted in duplicate.

### Metabolite profiling by UPLC–MS/MS

The samples were freeze–dried using a vacuum freeze dryer (Scientz–100F, Scientz, China) and subsequently pulverized into a powder using a grinder (MM 400, Retsch, Germany) at 30 Hz for a duration of 1.5 minutes. An accurate weight of 50 milligrams of the sample was measured (MS105DM) and mixed with 1200 μL of precooled 70% methanol extract at a temperature of –20°C. The mixture was subjected to vortexing for a period of 30 seconds at 30–minute intervals, resulting in a total of six vortexing sessions. Subsequently, the supernatant was collected following centrifugation at 12,000 × g for three minutes, and the sample was filtered through a microporous filter membrane with a pore size of 0.22 μm. The filtered sample was then placed in a vial for subsequent analysis.

The widely targeted metabolomic analysis was conducted in accordance with previously reported methods (Chen et al., 2013; Zhang et al., 2023). The data acquisition systems were primarily comprised of an ultra–high–performance liquid chromatography (UPLC) system (ExionLCTM AD, https://sciex.com.cn/) and a tandem mass spectrometry (MS/MS) system (Applied Biosystems 6500 QTRAP) (https://sciex.com.cn/). The analytical conditions were as follows, UPLC: column, Agilent SB-C18 (1.8 µm, 2.1 mm * 100 mm); The mobile phases comprised two distinct solvents: phase A, ultra–pure water with 0.1% formic acid, and phase B, acetonitrile with 0.1% formic acid. The elution gradient was set as follows: at 0.00 minutes, the proportion of phase B was 5%, over 9.00 minutes, it linearly increased to 95% and was maintained at that level for 1 minute, from 10.00 to 11.10 minutes, the proportion of phase B decreased back to 5% and was held at that level for 14 minutes. The flow rate was set at 0.35 mL/min, the column temperature was maintained at 40°C, and the injection volume was 2 μL.

The electrospray ionization (ESI)–triple quadrupole–linear ion trap (QTRAP) mass spectrometer was utilized for the analysis of the sample metabolites. The ESI source temperature was set to 500 °C, with an ion spray voltage (IS) of 5500 V for positive ion mode and –4500 V for negative ion mode. The ion source gas I (GSI), gas II (GSII), and curtain gas (CUR) pressures were set to 50, 60, and 25 psi, respectively, and the collision–induced dissociation parameters were configured to the highest level. The multiple reaction monitoring (MRM) mode was employed for the QQQ scan, with the collision gas (nitrogen) set to medium. Subsequently, further optimization of the declustering potential (DP) and collision energy (CE) was conducted for each MRM ion pair. A specific set of MRM ion pairs was monitored at each time interval, based on the metabolites eluted at that time (**Supplementary Table S1**; **Supplementary Figure S1**).

### Data processing

A combined UPLC–MS/MS detection platform was employed for the qualitative and quantitative analysis of metabolites. The metabolites were identified by means of their fragmentation patterns, retention times, and mass-to-charge ratios, with reference to a custom MWDB database. Secondary spectral information was employed for additional characterization. Metabolite quantification was achieved through multiple reaction monitoring (MRM) analysis, which employed triple quadrupole mass spectrometry. Once the metabolic mass spectrometry data from the various samples had been obtained, the chromatographic peaks of all substances were integrated based on peak area. Furthermore, the mass spectral peaks of the same metabolite across the different samples were integrated and normalized. By analyzing the proportions of metabolites, a comprehensive assessment of the distribution of major metabolites within the samples could be conducted. A principal component analysis (PCA) was conducted to gain preliminary insights into the overall metabolite differences among the samples and the degree of variation within each group. The data were subjected to unit variance scaling (UV), and a cluster heatmap was generated for all samples using R programming. Partial least squares discriminant analysis (PLS–DA) and orthogonal partial least squares discriminant analysis (OPLS–DA) were employed for the purpose of identifying differences between groups. In the case of two–group comparisons, differential metabolites were identified on the basis of variable importance in projection (VIP) values (VIP > 1), P–values (P < 0.05, Student’s t–test), and absolute log2 fold change (|Log_2_FC| ≥ 1.0). The VIP values were extracted from the OPLS–DA results, which included both score plots and permutation plots generated using the R package MetaboAnalystR. Prior to conducting OPLS–DA, the data underwent a log transformation (log2) and mean centering. To prevent overfitting, a permutation test with 200 permutations was conducted. The differential metabolites were then annotated using the KEGG compound database, and the resulting annotations were subsequently mapped to the KEGG pathway database. Subsequently, pathways containing significantly regulated metabolites were analyzed using metabolite set enrichment analysis (MSEA), with significance assessed through hypergeometric test P–values.

Heatmaps, Venn plots, and K–means graphs were generated using the R online software. A co–expression analysis was conducted using the WGCNA package in R, in accordance with the guidelines set forth in published tutorials (Langfelder and Horvath, 2008). A hierarchical clustering analysis was performed on the samples, with the distance between them calculated using the Euclidean metric based on the relative abundance of metabolite data. Network topology analysis confirmed the presence of a scale–free topology network with a defined soft–thresholding power of 11. The dynamic tree cutting algorithm identified a total of nine modules, with parameters set to a minimum module size of 30 and a merge cut height of 0.25.

### Statistical analysis

All samples were composed of three replicates, and the means and standard errors were calculated from these data. The statistical analyses were conducted using GraphPad Prism software (Version 8.3), and the results are presented as mean ± SD. Student’s t-test was used for two-group comparisons and one-way ANOVA followed by Duncan’s test for multiple group comparisons.

## Results

### Phenotypic characterization of inflorescences of 11 different macadamia varieties

We selected 11 macadamia varieties with a wide range of variation in inflorescence phenotypes, and these samples represented different morphological characteristics of macadamia inflorescences, including inflorescence length, color, distribution density of flowers on the inflorescence, and overall shape of the inflorescence (**Figure 1**). Each inflorescence is composed of numerous small flowers, which are densely arranged in racemes. The inflorescences exhibit a length range of 8 to 20 cm, with pedicels measuring 3–4 mm in length and bracts that are nearly ovoid in shape. The filaments are relatively short, and the anthers are approximately 1.5 mm in length. At full bloom, the flowers commonly display pale yellow or white hues. The length and color of the inflorescence are significant indicators of Macadamia traits, and as illustrated in **Figure 1**, these features vary across cultivars, likely influenced by both genetic and environmental factors.

### Measurement of indicators related to inflorescence color formation

**Figure 2:**
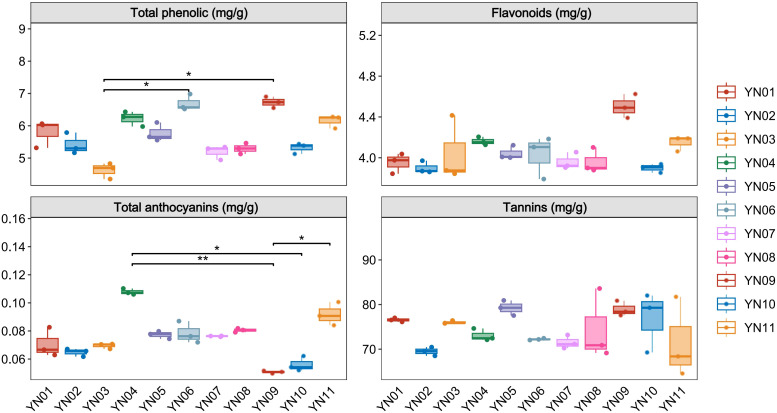
Total phenolic, flavonoids, total anthocyanins and tannins in different macadamia inflorescence samples. One-way ANOVA with Duncan’s test was applied for multiple group comparisons. The data represent mean ± SD, n=3. * indicates statistically significant difference in values (p< 0.05); ** indicates statistically significant difference in values (p< 0.01).

To investigate the factors contributing to the formation of different inflorescence colors in Macadamia integrifolia, we analyzed the contents of phenolic compounds, flavonoids, tannins, and total anthocyanins in the inflorescences of eleven cultivars (YN01–YN11). Results indicated variability in the levels of these compounds across the samples (**Figure 2**). Total phenolic content exhibited notable variation, with YN09 and YN06 showing higher values and YN03 the lowest, demonstrating significant differences between groups (p < 0.05). Flavonoid content also varied, with YN09 showing the highest concentration. For total anthocyanins, YN04 was significantly higher than YN09 (p < 0.01) and YN10 (p < 0.05). Tannin content displayed trends of variation among samples, with higher levels observed in YN01, YN03, YN05, YN09, and YN10, although inter–group differences were not statistically significant (p = 0.132). These compounds may play crucial roles in influencing the formation of different inflorescence colors in Macadamia cultivars.

### Overall characterization of non–volatile metabolites

**Figure 3:**
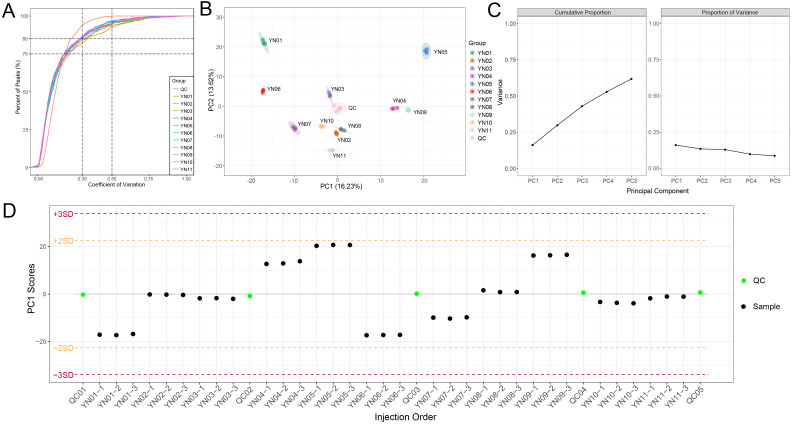
Overall characterization of nonvolatile metabolites. **(A)** Coefficient of variation (CV) distribution for metabolite abundances across samples. Over 75% of metabolites exhibited CV < 0.3, indicating high reproducibility. **(B)** Principal component analysis for all samples. Each point in the graph represents a sample, and samples from the same group are represented using the same color. **(C)** The variance explained by each principal component, including the cumulative proportion and the proportion of variance of the first five principal components. **(D)** Process control for principal component univariate statistics. Each point in the graph represents a sample, and the horizontal coordinate is the order of sample detection. The points in the graph will show up and down fluctuations due to changes in the instrument status, and generally the PC1 Scores of the quality control samples are within the range of plus or minus 3 standard deviations as the normal range.

A broad–targeted metabolomic approach was utilized to examine the non–volatile metabolites present in the YN01–YN11 cultivars. In light of the considerable number of sample groups included in this study, it was imperative to assess the extent of outliers. The coefficient of variation (CV), defined as the ratio of the standard deviation to the mean of the original data, was employed to reflect the dispersion of the data. The empirical cumulative distribution function (ECDF) was employed to analyze the frequency of substances exhibiting CV values below a reference threshold. The results demonstrated that over 75% of metabolites in all samples exhibited CV values below 0.3, indicating high data stability (**Figure 3A**). A PCA was conducted on the samples, inclusive of the quality control samples, to evaluate both the inter–group metabolic distinctions and the intra–group variability. The results of the PCA revealed distinct separation trends among the groups, accompanied by high intra–group consistency (**Figure 3B**). Principal components PC1 and PC2 accounted for 16.23% and 13.62% of the variation, respectively, while the top five principal components collectively explained 61.77% of the total variation (**Figure 3C**). Furthermore, PCA was employed to monitor the quality control (QC) samples via ion peaks detected in each sample, thereby confirming instrument stability as evidenced by PC1 scores that remained within ±2 standard deviations (SD) (**Figure 3D**). These findings indicate high repeatability and reliability of the metabolomic data, as well as clear discrimination among groups.

**Figure 4:**
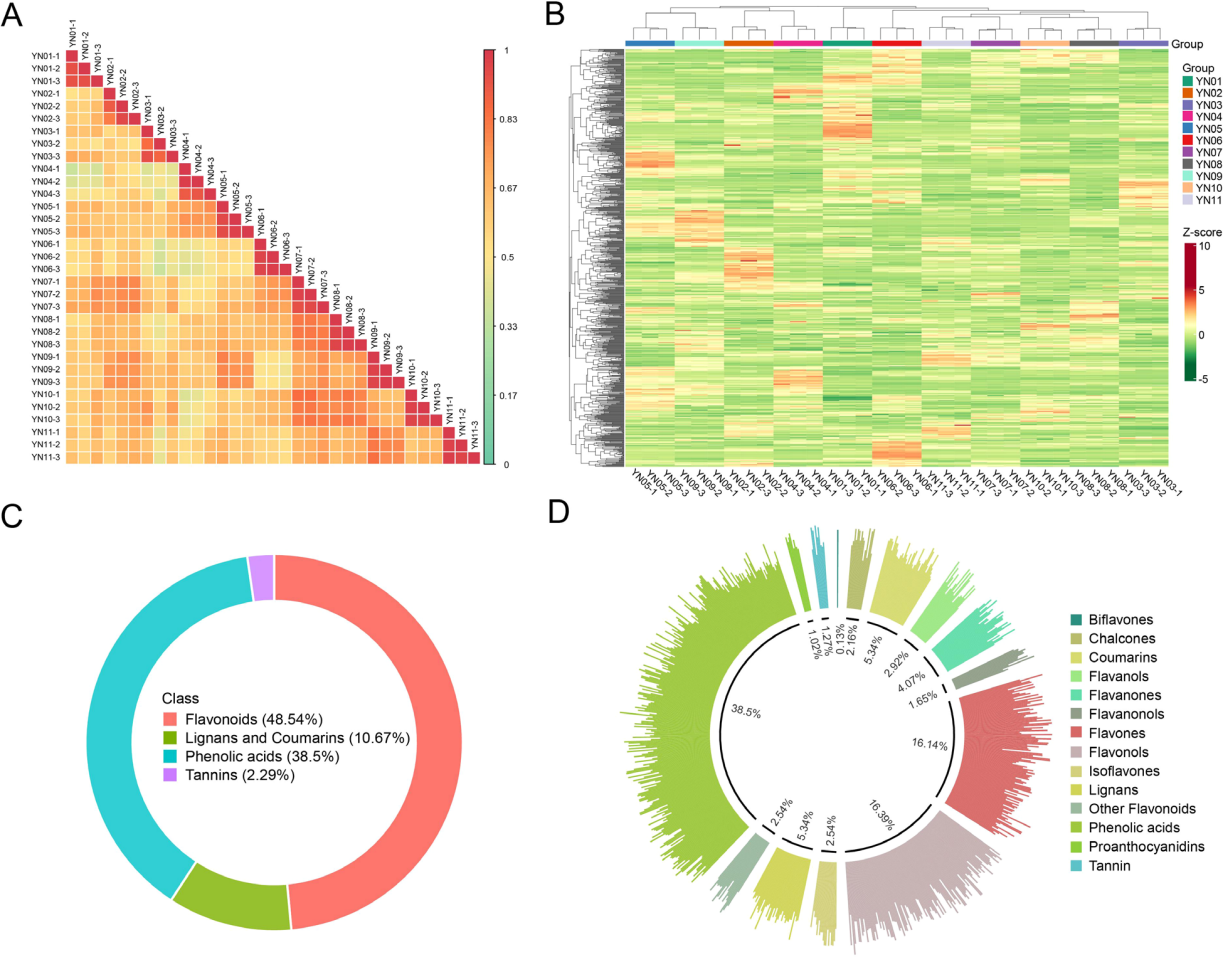
Correlation analysis of samples and identification of metabolites. Pearson correlation heatmaps for all samples **(A)**, and clustered heatmaps for all metabolites **(B)**. Primary **(C)** and secondary **(D)** classification of all identified metabolites.

Pearson correlation analysis and hierarchical clustering yielded results consistent with the PCA findings, demonstrating high intra–2group consistency and clear inter–group separation, thereby further confirming the accuracy of the data in this study (**Figures 4A, B**). A total of 787 metabolites were identified across the eleven sample groups, comprising 38 flavonoids (48.5%), 303 phenolic acids (38.5%), 84 lignans and coumarins (10.7%), and 18 tannins (2.3%) (**Figure 4C**). Among secondary metabolite classifications, the most prevalent were phenolic acids (303, 38.5%), followed by flavonols (129, 16.4%) and flavones (127, 16.1%). Furthermore, coumarins (42, 5.3%) and lignans (42, 5.3%) each constituted over 5% of the identified metabolites (**Figure 4D**).

### Identification of differential metabolites of different colored inflorescences

**Figure 5:**
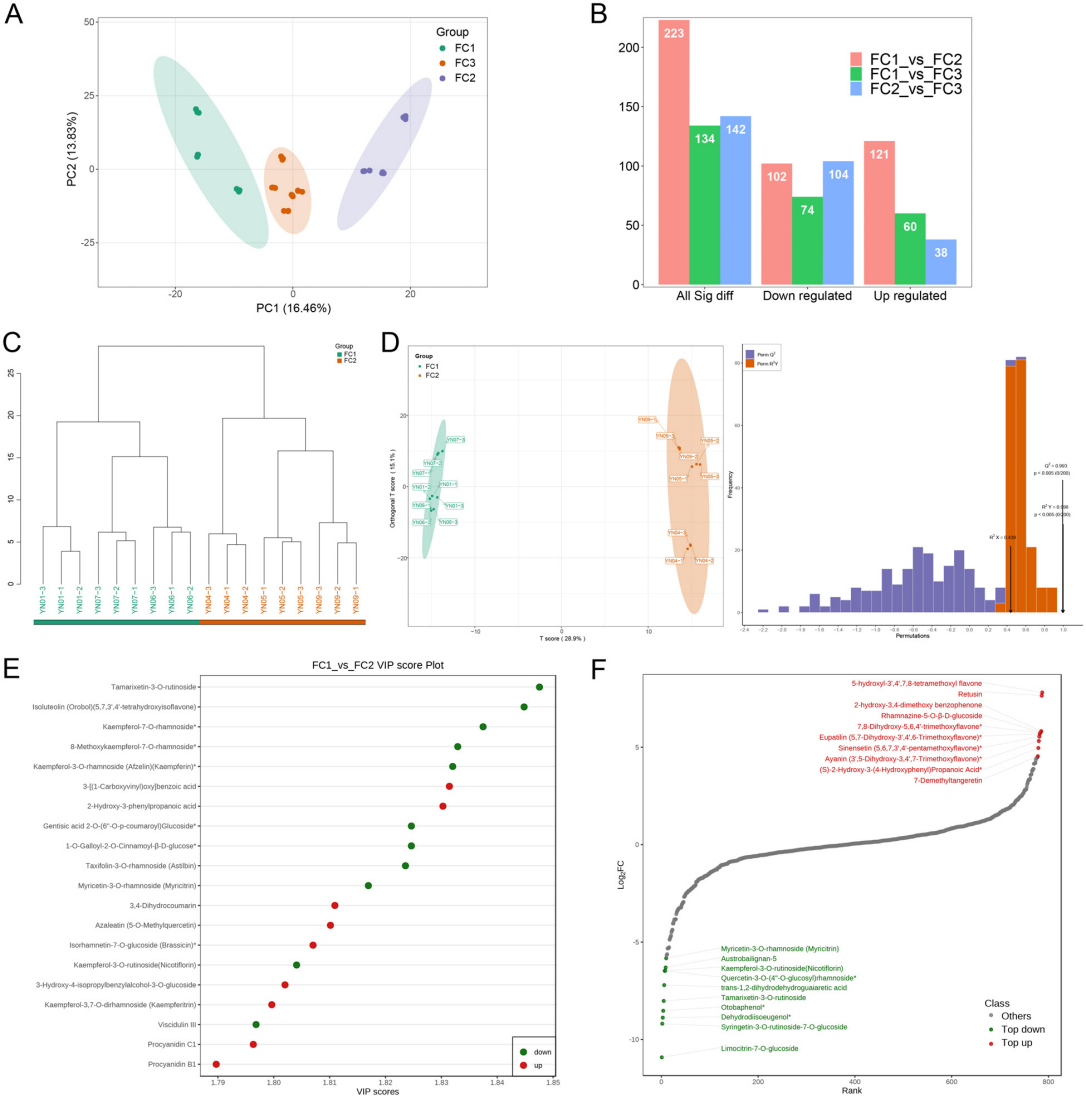
Screening and identification of differential metabolites. **(A)** PCA plot after regrouping based on phenotype and metabolite identification. **(B)** Histogram of the number of differential metabolites in the different comparison groups. **(C)** Hierarchical clustering plot of samples from the FC1 and FC2 groups. **(D)** OPLS-DA diagrams for FC1 and FC2 groups. Horizontal coordinates denote predicted principal components and vertical coordinates denote orthogonal principal components, which indicate inter- and intra-group disparities, respectively. The right-hand panel shows the validation of the OPLS-DA model. The horizontal coordinates represent the model R^2^Y, Q^2^ values, and the vertical coordinates are the frequency of the model classification effects appearing in 200 random permutation experiments. The orange color in the figure represents the random permutation model R^2^Y, the purple color represents the random permutation model Q^2^, and the values represented by the black arrows are the R^2^X, R^2^Y, and Q^2^ values of the original model. **(E)** Map of the top 20 differential metabolites with the largest VIP values in FC1 vs FC2. **(F)** Plot of the dynamics of the top 10 up- and down-regulated metabolite contents with the largest fold change in FC1 vs FC2.

The phenotypic results and the PCA clustering data of metabolites from the collected samples were used to classify the eleven sample groups into three major flower color categories. The first flower color category (FC1) encompasses YN01, YN06, and YN07, the second (FC2) includes YN04, YN05, and YN09, and the third (FC3) is comprised of YN02, YN03, YN08, YN10, and YN11 (**Figure 5A**). The identification of differential metabolites was conducted using the following thresholds: variable importance in projection (VIP) score of 1 or greater, fold change (FC) of 2 or less or greater than 0.5, and a P–value of less than 0.05. The results demonstrated the presence of 223 differential metabolites in the FC1 vs. FC2 comparison, with 121 exhibiting increased expression and 102 displaying decreased expression. In the comparison between FC1 and FC3, 134 differential metabolites were identified, comprising 60 that were up–regulated and 74 that were down–regulated. The FC2 vs. FC3 comparison yielded 142 differential metabolites, of which 38 were up–regulated and 104 were down–regulated (**Figure 5B**). The results of the hierarchical clustering and OPLS–DA analysis demonstrated a clear distinction between the groups, with 200 permutation tests on the OPLS–DA model confirming high reliability (Q^2^ = 0.993, R^2^Y = 0.998, both P < 0.005). This indicates that the model was not overfitted and that the group classification was appropriate (**Figures 5C, D**). The results of the FC1 vs. FC3 and FC2 vs. FC3 comparisons are consistent with those of the previous analysis, providing further support for the rationale behind these differential groupings (**Supplementary Figures S2**, **S3**). The top 20 metabolites with the highest VIP values in the OPLS–DA model, comprising 11 down–regulated and 9 up–regulated metabolites, are highlighted in **Figure 5E**. The metabolites with the largest VIP values among the down–regulated metabolites were Tamarixetin–3–O–rutinoside (1.848), Isoluteolin (Orobol) (5,7,3’,4’–tetrahydroxyisoflavone) (1.845) and Kaempferol–7–O– rhamnoside* (1.837). The metabolites with the largest VIP values among the up–regulated metabolites were 3–[(1–Carboxyvinyl)oxy]benzoic acid (1.831) and 2–Hydroxy–3–phenylpropanoic acid (1.83), respectively. To more clearly and intuitively demonstrate the overall metabolic differences, the metabolites in the comparison group were ranked from smallest to largest according to the magnitude of the multiplicity value of the differences. A dynamic distribution plot of the differences in metabolite content was then generated, and the top 10 metabolites in the up–regulated and down–regulated metabolites were labeled, revealing that the highest up–regulated metabolites (log_2_FC) were 5–hydroxyl–3’,4’,7,8– tetramethoxyl flavone (7.81), Retusin (7.65) and Rhamnazine–5–O–β–D–glucoside (5.76). The metabolites with the highest down–regulation were Limocitrin–7–O–glucoside (–10.92), Syringetin–3–O–rutinoside–7–O–glucoside (–9.19) and Dehydrodiisoeugenol* (–8.88) (**Figure 5F**). Subsequently, the metabolites that exhibited differential expression in the remaining two comparison groups were subjected to the same analytical approach (**Supplementary Figures S2**, **S3**).

### Enrichment analysis of differential metabolites

**Figure 6:**
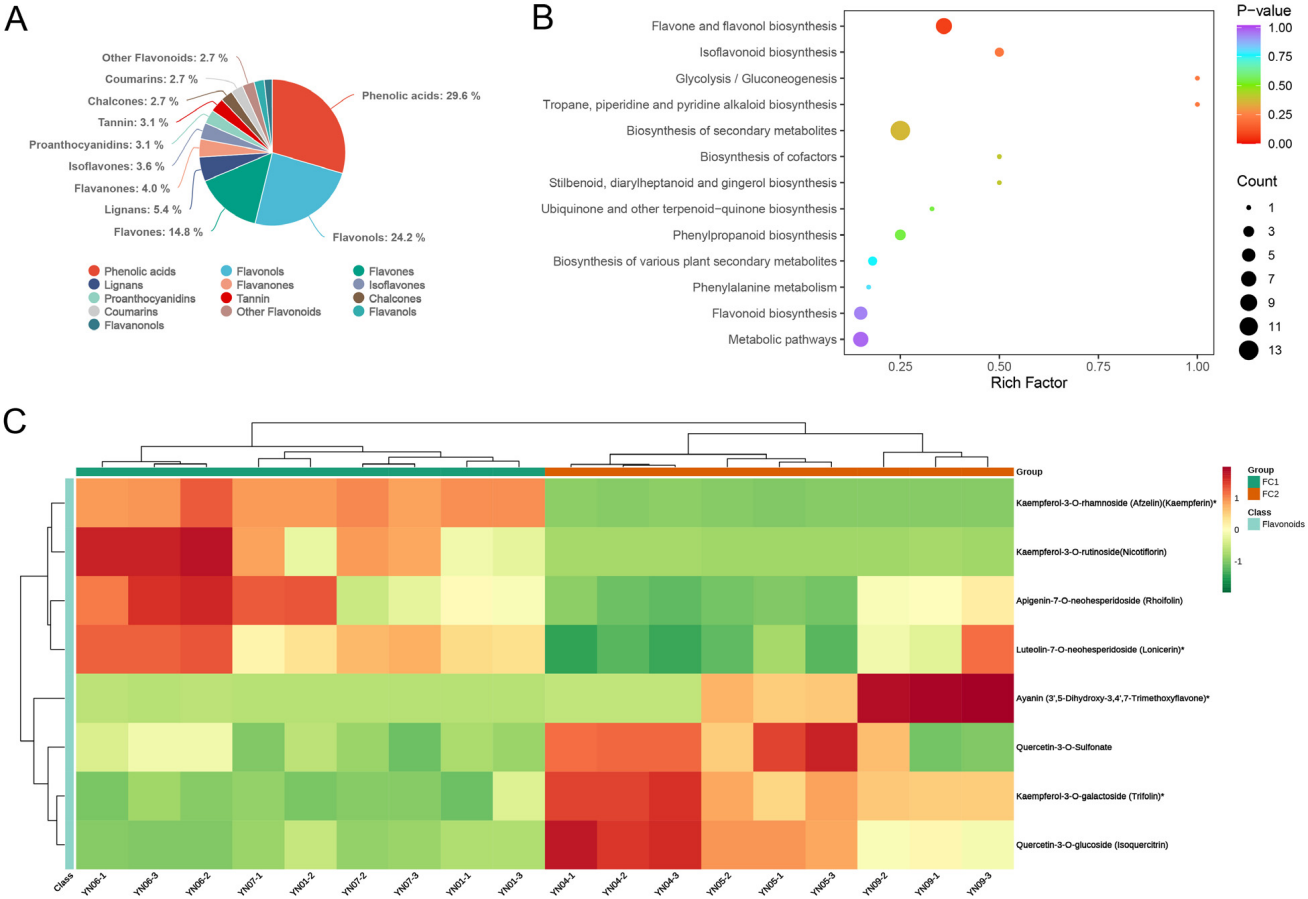
Identification and enrichment analysis of differential metabolites. **(A)** Pie chart of secondary categorization of differential metabolites in FC1 vs FC2. **(B)** The KEGG enrichment analysis of differential metabolites. The horizontal coordinate indicates the corresponding Rich Factor for each pathway, the vertical coordinate is the pathway name, the color of the dot reflects the P-value, and the size of the dot represents the number of differential metabolites. **(C)** Clustered heat map of the expression abundance of eight differential metabolites in Flavone and flavonol biosynthesis in the two groups. The horizontal coordinate is the name of the sample, the vertical coordinate is the differential metabolite, and the different colors are the colors that are filled with the different values obtained from the standardization treatment of the different relative contents (red for high content, green for low content).

Subsequently, an additional analysis was conducted on the differential metabolites identified in each comparison group. Initially, the metabolites were classified into secondary categories. In all three comparison groups, the majority of differential metabolites were concentrated in the categories of phenolic acids, flavonols, and flavones, collectively accounting for approximately 70% of the metabolites. It is noteworthy that in the FC1 vs. FC2 and FC1 vs. FC3 comparisons, phenolic acids constituted the largest proportion of differential metabolites, with 66 and 62, respectively, representing 29.6% and 46.3% of the total differential metabolites. This indicates that phenolic acids play a pivotal role in the development of inflorescence color in the FC1 group (**Figure 6A**; **Supplementary Figure S4A**). The KEGG enrichment analysis revealed that in the FC1 vs. FC2 comparison, the differential metabolites were predominantly enriched in pathways associated with flower color formation, including flavone and flavonol biosynthesis, phenylpropanoid biosynthesis, flavonoid biosynthesis, and isoflavonoid biosynthesis. Among these, the pathway with the highest enrichment significance (lowest p–value) was flavone and flavonol biosynthesis (**Figure 6B**). A heatmap of cluster analysis displays the expression abundance of eight differential metabolites involved in this pathway between the two groups. In FC1, the abundance of Kaempferol–3–O–rhamnoside (Afzelin)(Kaempferin)*, Kaempferol–3–O–rutinoside (Nicotiflorin), Apigenin–7–O–neohesperidoside (Rhoifolin), and Luteolin–7–O–neohesperidoside (Lonicerin)* was significantly higher, while Ayanin (3’,5–Dihydroxy–3,4’,7–Trimethoxyflavone)*, Quercetin–3–O–Sulfonate, Kaempferol–3–O–galactoside (Trifolin)*, and Quercetin–3–O–glucoside (Isoquercitrin) were more abundant in FC2, indicating these metabolites may play important roles in the formation of different flower colors between FC1 and FC2 (**Figure 6C**). Similarly, the differential metabolites identified in FC1 vs. FC3 were primarily enriched in the pathways of biosynthesis of various plant secondary metabolites, phenylalanine metabolism, and biosynthesis of amino acids, with the most significant enrichment observed in biosynthesis of various plant secondary metabolites (p = 0.037). In the comparison between FC2 and FC3, the differential metabolites were found to be significantly enriched in the pathways of secondary metabolite biosynthesis and flavone and flavonol biosynthesis, with p–values of 0.0262698 and 0.02627, respectively (**Supplementary Figure S4B**).

### Identification of key metabolites involved in flower color formation

**Figure 7:**
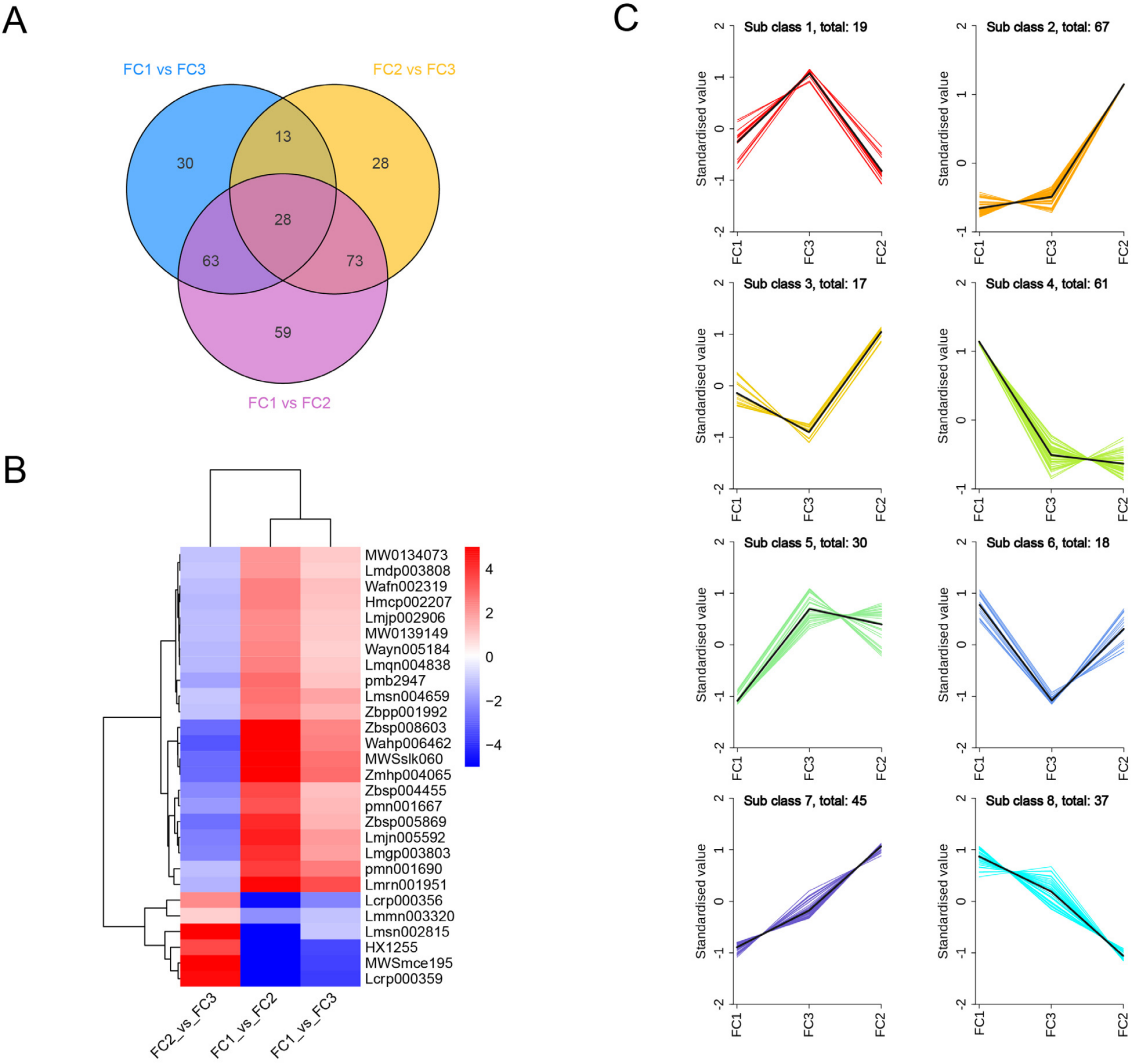
Identification of key metabolites involved in inflorescence color formation. **(A)** Venn diagrams of differential metabolites in the three comparison groups. **(B)** Clustering heatmap of the 28 differential metabolites present in the three comparison groups. **(C)** K-Means cluster analysis of all differential metabolites. Horizontal coordinates indicate sample groupings and vertical coordinates indicate standardized relative metabolite levels.

To further identify the key metabolites involved in the formation of inflorescence color in *Macadamia integrifolia*, we took the intersection of the differential metabolites across the three comparison groups. The results demonstrated that 28 metabolites were consistently identified. Among the identified metabolites, 22 exhibited a significant up–regulation in the FC1 vs. FC2 and FC1 vs. FC3 comparison groups, while displaying a notable down–regulation in the FC2 vs. FC3 group. In contrast, six metabolites exhibited an inverse trend, indicating that these metabolites may play a pivotal role in the development of diverse inflorescence colors in *Macadamia* (**Figures 7A, B**). In terms of primary classification, 16 of these differential metabolites belong to flavonoids, five to lignans and coumarins, four to phenolic acids, and three to tannins. The primary pathways involved include flavone and flavonol biosynthesis (ko00944) and biosynthesis of secondary metabolites (ko01110). Detailed information on these 28 metabolites is provided in **Table 1**.

**Table 1:** Metabolites co–identified in the three differential comparison groups.

Compounds	FC1 vs FC2			FC1 vs FC3			FC2 vs FC3		
	log2FC	p–value	VIP	log2FC	p–value	VIP	log2FC	p–value	VIP
3–Hydroxy–4–isopropylbenzylalcohol–3–O–glucoside	3.80	0.00	1.80	2.63	0.00	2.13	–1.17	0.00	1.79
(S)–2–Hydroxy–3–(4–Hydroxyphenyl)Propanoic Acid*	4.96	0.00	1.35	3.50	0.00	1.49	–1.46	0.01	1.35
2,6–Dimethoxy–4–(7–methoxy–3–methyl–5–prop–1–enyl–2,3–dihydro–1–benzofuran–2–yl)phenol	–4.74	0.01	1.40	–2.37	0.03	1.16	2.37	0.05	1.19
1,6–Di–O–galloyl–2–O–Feruloyl–β–D–glucose	2.81	0.00	1.54	1.77	0.00	1.80	–1.04	0.01	1.44
Dehydrodiisoeugenol*	–8.88	0.01	1.45	–3.68	0.02	1.13	5.20	0.03	1.65
Isorhamnetin 3–galactoside	2.17	0.00	1.66	1.10	0.01	1.01	–1.07	0.00	1.45
Otobaphenol*	–8.53	0.01	1.45	–3.70	0.02	1.13	4.83	0.03	1.36
Ayanin (3’,5–Dihydroxy–3,4’,7–Trimethoxyflavone)*	5.32	0.01	1.54	2.46	0.02	1.53	–2.86	0.01	1.60
Rhamnetin–3–O–Glucoside*	2.30	0.00	1.74	1.09	0.01	1.11	–1.22	0.00	1.63
Nepitrin*	2.30	0.00	1.74	1.09	0.01	1.11	–1.22	0.00	1.63
Eupatilin (5,7–Dihydroxy–3’,4’,6–Trimethoxyflavone)*	5.68	0.01	1.57	2.86	0.01	1.63	–2.82	0.01	1.57
Catechin–catechin–catechin	2.94	0.00	1.77	1.26	0.05	1.10	–1.68	0.00	1.87
Kaempferol–3–O–rutinoside(Nicotiflorin)	–6.48	0.00	1.80	–1.00	0.04	1.31	5.48	0.00	1.62
7,4’–Dihydroxy–3,5,3’–trimethoxyflavone	4.19	0.01	1.40	1.49	0.02	1.01	–2.71	0.02	1.48
Tricin–5,7–O–diglucoside*	3.59	0.01	1.42	1.35	0.02	1.06	–2.24	0.01	1.43
7,8–Dihydroxy–5,6,4’–trimethoxyflavone*	5.75	0.01	1.56	2.89	0.01	1.67	–2.86	0.01	1.59
trans–1,2–dihydrodehydroguaiaretic acid	–7.21	0.02	1.45	–3.55	0.03	1.26	3.66	0.01	1.70
3’–O–Methylellagic acid 4–O–(2’’–dihydroxybenzoylglucoside)	2.38	0.00	1.61	1.02	0.01	1.08	–1.36	0.01	1.55
Procyanidin B4	3.39	0.00	1.72	1.46	0.00	1.68	–1.93	0.00	2.03
Cirsilineol (4’,5–Dihydroxy–3’,6,7–trimethoxyflavone)	4.47	0.01	1.52	2.02	0.00	1.62	–2.45	0.01	1.49
4–O–(3’–O–alpha–D–Glucopyranosyl)caffeoylquinic acid	2.50	0.00	1.63	1.28	0.00	1.90	–1.23	0.00	1.81
3–O–Methylellagic acid	2.50	0.00	1.26	1.14	0.00	1.40	–1.36	0.00	2.31
Rhamnazine–5–O–β–D–glucoside	5.78	0.03	1.67	2.50	0.00	1.98	–3.27	0.05	1.79
Azaleatin (5–O–Methylquercetin)	2.09	0.00	1.81	1.04	0.00	1.72	–1.05	0.00	1.89
Isorhamnetin–3–O–Glucoside*	2.63	0.00	1.75	1.54	0.00	1.55	–1.08	0.00	1.57
Isorhamnetin–7–O–glucoside (Brassicin)*	2.57	0.00	1.81	1.26	0.00	1.40	–1.31	0.00	1.75
5’–Methoxyisolariciresinol–9’–O–xyloside	–2.14	0.00	1.12	–1.08	0.01	1.26	1.05	0.04	1.26
5,7,4’–Trihydroxy–6,8–dimethoxyisoflavone–7–O–galactoside–rhamnose	4.18	0.01	1.19	1.88	0.01	1.29	–2.31	0.01	1.46

To examine the relative content variation trends of metabolites across different groups, we applied unit variance scaling (UV) to all identified differential metabolites based on the specified selection criteria and subsequently performed K–means clustering analysis. The results indicated the presence of eight discernible trends among the differential metabolites. Class 1 represents the characteristic metabolites associated with the inflorescence color in the FC3 group, comprising 19 metabolites, 12 of which are classified as phenolic acids, accounting for 63.2%. In contrast, Class 2, which encompasses 67 metabolites, is representative of the inflorescence color in FC2, with flavones accounting for 32.8%. Similarly, Class 4 consists of 61 metabolites representative of the inflorescence color in FC1, with phenolic acids comprising 50.8%, which is consistent with previous findings. It is noteworthy that two intriguing clusters, Class 7 and Class 8, illustrate a gradual metabolic transition from FC1 to FC2 in inflorescence color, with flavonoids representing the predominant metabolites in both classes at 60% and 78.3%, respectively (**Figure 7C**). This indicates that the alteration of inflorescence color in *Macadamia integrifolia* is predominantly governed by flavonoids. Further analysis revealed that five metabolites from the 28 differential metabolites shared with class 4 are categorized as lignans rather than flavonoids or phenolic acids. Furthermore, an additional ten metabolites were identified as common between the aforementioned 28 differential metabolites and Class 2. Of these, nine were categorised as flavonoids, while the remaining one, Tannin, was identified as Procyanidin B4. Lastly, 12 metabolites from the 28 were also identified in Class 7, with only one, Kaempferol–3–O–rutinoside (Nicotiflorin), shared with Class 8 (**Supplementary Figure S5**). These findings suggest that these metabolites are integral to the development of diverse inflorescence colors.

**Figure 8:**
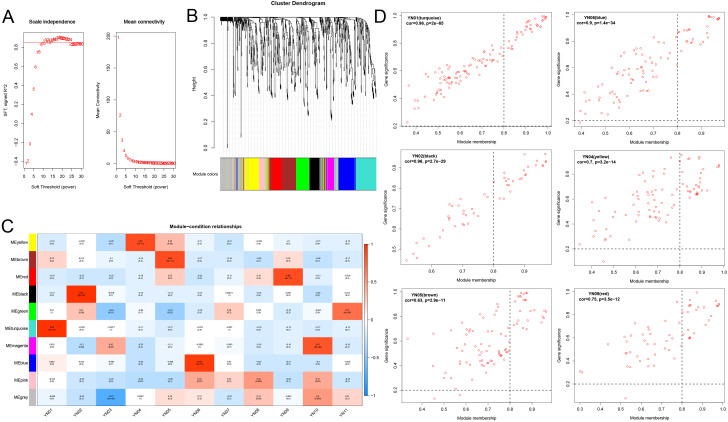
WGCNA analysis screens for key metabolites in macadamia inflorescence color formation. **(A)** Soft thresholds and average connectivity for WGCNA analysis. **(B)** The cluster dendrogram of WGCNA. **(C)** Heatmap of correlations between modules and samples, where the horizontal coordinates represent different samples and the vertical coordinates represent different modules. **(D)** Scatterplot of the top 6 modules with the highest correlation between modules and samples.

To further identify the key metabolites involved in the formation of inflorescence color in *Macadamia integrifolia*, a WGCNA analysis was employed (**Figure 8**). Based on the metabolite abundance data from this study, the platform threshold was set at 0.85, with a soft threshold of 11 (**Figure 8A**). A dynamic hybrid cutting algorithm was employed to generate gene modules and to optimize merged gene modules, with a minimum merging distance of 0.25. The minimum number of metabolites per module (minModuleSize) was set at 30, resulting in a total of 10 distinct modules (**Figure 8B**). Subsequently, the correlation between modules and groups was calculated, and a correlation heatmap was plotted in order to identify the modules that were most significantly associated with the groups. The results demonstrated that the correlation between modules and groups did not exhibit a similar degree of alignment with the PCA and phenotypic results. The six modules exhibiting the highest correlations with the groups were selected for further analysis (cor > 0.87, p < 7e–11). TN01, TN02, YN04, YN05, YN06, and YN09 exhibited a markedly positive correlation with the MEturquoise, MEblack, MEyellow, MEbrown, MEblue, and MEred modules, respectively (**Figure 8C**). Scatter plots of the kME values of these modules against the results of the metabolite significance analysis were employed to identify key metabolites (**Figure 8D**). The intersection of metabolites within each module and the differential metabolites identified through K–means clustering yielded significantly differential metabolites for each module. Specifically, the YN04 (yellow) module exhibited 51 shared differential metabolites, while the YN05 (brown), YN01 (turquoise), YN06 (blue), YN09 (red), and YN02 (black) modules displayed 42, 42, 38, 28, and 16 differential metabolites, respectively (**Supplementary Figure S6A**). A KEGG enrichment analysis of the differential metabolites (totaling 121) belonging to the FC2 module revealed significant enrichment in the flavone and flavonol biosynthesis pathway, with the lowest p–value. Four flavonoids–Ayanin (3’,5–Dihydroxy–3,4’,7–Trimethoxyflavone)*, Quercetin–3–O–glucoside (Isoquercitrin), Quercetin–3–O–Sulfonate, and Luteolin (5,7,3’,4’–Tetrahydroxyflavone) are likely to play a significant role in the color formation of FC2 (**Supplementary Figure S6B**). In contrast, metabolites in FC1 exhibited greater enrichment in the biosynthesis of various plant secondary metabolites and phenylpropanoid biosynthesis pathways. Additionally, four metabolites were identified in the flavonoid biosynthesis pathway (**Supplementary Figure S6C**).

**Figure 9:**
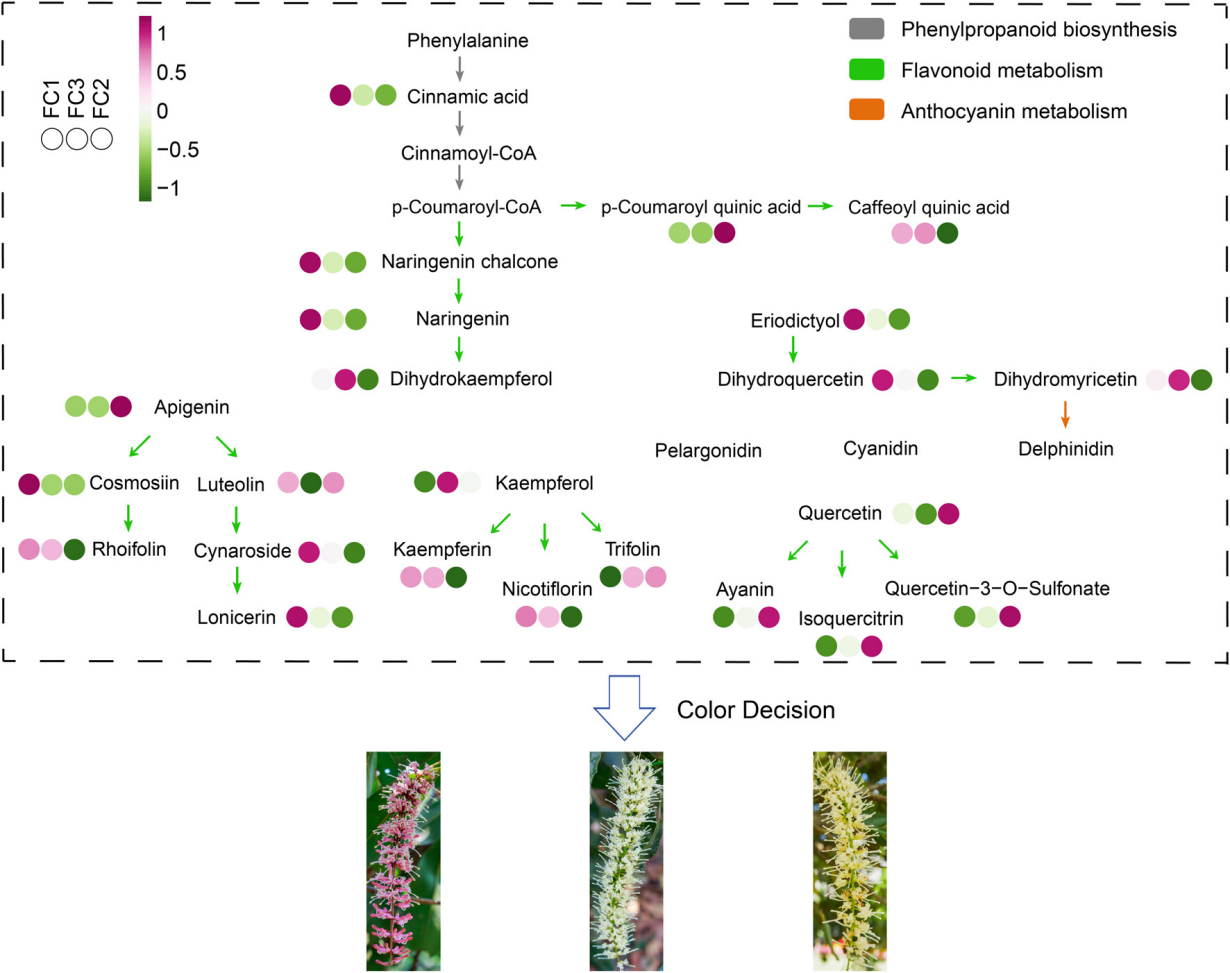
Schematic diagram of the metabolic pathway mechanisms involved in macadamia inflorescence color determination. Different colored arrows indicate different metabolic pathways.

By integrating the insights from these diverse analytical techniques, our findings revealed that the flavonoid biosynthesis pathway is a pivotal factor in the development of inflorescence color in *Macadamia integrifolia*. Based on the metabolite abundance obtained in this study, a schematic diagram was constructed to illustrate the mechanisms of phenylpropanoid biosynthesis, flavonoid metabolism, and anthocyanin metabolism in the synthesis of different inflorescence colors in *Macadamia integrifolia* (**Figure 9**).

## Discussion

Macadamia nuts are highly regarded as a nutritionally valuable crop due to their richness in unsaturated fatty acids, proteins, vitamins, and other bioactive compounds, which contribute to their recognized health benefits and high nutritional value (Du et al., 2010). To date, China has become the largest and fastest–growing producer of macadamia nuts worldwide. Research aimed at enhancing macadamia nut yield has predominantly focused on disease prevention (Li et al., 2022a, b) and crop cultivation techniques (Russell et al., 2018), with relatively little exploration into the molecular mechanisms by which floral development affects fruit quality and yield (Wagner, 2017). As a crucial reproductive organ, flowers play an essential role in determining reproductive success and yield. In this study, an analysis was conducted on 11 macadamia inflorescence samples exhibiting distinct color phenotypes (YN01–YN11). The results demonstrated notable variations in the observable phenotypes (**Figure 1**) and traits associated with floral coloration, including total phenolic content, flavonoids, tannins, and anthocyanins (**Figure 2**). Furthermore, a comprehensive analysis employing widely targeted metabolomics identified a total of 787 metabolites, including 382 flavonoids (48.5%), 303 phenolic acids (38.5%), 84 lignans and coumarins (10.7%), and 18 tannins (2.3%) (**Figure 4**). Among the secondary metabolite categories, the most prevalent were phenolic acids (303, 38.5%), followed by flavonols (129, 16.4%) and flavonoids (127, 16.1%), which is consistent with the findings of previous studies (Wang et al., 2023). Principal component analysis (PCA) based on phenotypic and metabolite profiles yielded a grouping of all samples into three clusters, designated as FC1, FC2, and FC3, respectively. A differential metabolite analysis revealed 223 differentially abundant metabolites between FC1 and FC2, 134 between FC1 and FC3, and 142 between FC2 and FC3 (**Figures 5A, B**). The KEGG enrichment analysis of these differential metabolites indicated that the majority were concentrated in the categories of phenolic acids, flavonols, and flavonoids. In comparisons of FC1 vs. FC2 and FC1 vs. FC3, the largest proportion of differential metabolites were identified as phenolic acids, which were primarily enriched in pathways associated with floral coloration, including flavonoid and flavonol biosynthesis and phenylpropanoid biosynthesis. These findings indicate that phenolic acids are integral to the floral color development of FC1, emphasizing the intricate and specific metabolic pathways involved in flower coloration. Further analysis indicated that flavonoids predominantly regulate the color variation observed in macadamia inflorescences. Analytical approaches, including K–means clustering and weighted gene co–expression network analysis (WGCNA), revealed distinct distribution trends and modular correlations for color–related metabolites across the diverse floral color categories. For instance, phenolic acids were more prevalent among the characteristic metabolites of FC3, flavonoids were predominant in FC2, and phenolic acids were once again significant in FC1, where they acted synergistically with flavonoids in the color metabolic transition from FC1 to FC2 (**Figure 7**). WGCNA identified modules that were significantly associated with specific floral color groups. KEGG enrichment of differential metabolites in these modules revealed significant enrichment in flavonoid and flavonol biosynthesis pathways (**Figure 8**). Based on the aforementioned findings, a conceptual framework was constructed to illustrate the mechanism underlying the macadamia floral coloration. This framework encompasses pathways such as phenylpropanoid biosynthesis, flavonoid metabolism, and anthocyanin metabolism. The model illustrates the pivotal role of flavonoid biosynthesis in the formation of macadamia inflorescence color (**Figure 9**).

### The significant role of floral color in the reproduction of macadamia

A single macadamia tree has the potential to produce up to 3,500 racemose inflorescences in a single year (Moncur et al., 1985), with each inflorescence containing between 100 and 300 flowers. However, macadamia exhibits a notably low fruit–to–flower ratio, a characteristic common among Proteaceae species (Ayre and Whelan, 1989). It is typical for less than 2% of macadamia flowers to develop into mature fruits. Macadamia flowers are protandrous and hermaphroditic, displaying partial self–incompatibility (Trueman, 2013). Cross–pollination between different cultivars has been shown to positively impact yield by increasing nut retention and maximizing nut weight (Langdon et al., 2020). Previous studies have demonstrated that, in comparison to self–pollination with pollen from the same cultivar, cross–pollination with pollen from different cultivars promotes greater pollen tube growth and higher rates of fruit set in developing nuts (Sedgley et al., 1990). Effective pollination of macadamia is facilitated by honeybees (*Apis mellifera*) and stingless bees (*Tetragonula* spp., primarily *T. carbonaria*) (Evans et al., 2021). Flower color and volatile organic compounds (VOCs) serve as the primary mechanisms for attracting honeybees and other insect pollinators (Peach et al., 2020; Dudareva et al., 2013), which in turn influence fruit set and nut yield. Flower color plays a significant role in influencing pollinator behavior (Ida and Kudo, 2010). The sensitivity of different insect species to color varies considerably. Floral colors with high brightness and contrast are particularly effective in attracting key pollinators, such as bees and butterflies. This enhances pollen transfer and improves pollination success. A positive correlation has been demonstrated between the saturation and brightness of floral color and the frequency of insect visitation (Shrestha et al., 2019). Additionally, variations in flower color may serve as an indicator of plant responses to environmental conditions. In the context of adverse growth conditions, such as limited light or nutrient deficiencies in the soil, alterations in flower coloration may assist plants in optimizing resource allocation and enhancing reproductive success (Tai et al., 2020). Moreover, effective pollination not only enhances fruit set rates but also optimizes fruit development and quality.

### The role of flavonoids in the inflorescence color variation of macadamia

Extensive research has demonstrated that carotenoids, flavonoids, and alkaloids are the primary pigments responsible for floral coloration (Zhao and Tao, 2015; Ma et al., 2018). Of these, carotenoids and flavonoids have been the subject of particular interest due to their pervasive distribution in plants and their capacity to generate vivid petal colors. Carotenoids contribute to a range of hues, from bright red to orange and yellow, while flavonoids, as major secondary metabolites in plants, generate colors ranging from pale yellow to purple, depending on their specific type (Zhu et al., 2019). Prior research has identified flavonoids as pivotal metabolites in the determination of macadamia inflorescence coloration. Specifically, compounds such as delphinidin–3–O–glucoside, petunidin–3–O–arabinoside, delphinidin–3–O–arabinoside, and cyanidin–3–O–arabinoside are postulated to play a role in floral pigmentation and the downstream formation of VOCs (volatile organic compounds), which aid in attracting pollinators, thereby enhancing fruit set and yield (Wang et al., 2023). Our analysis indicates that three metabolic pathways, phenylpropanoid biosynthesis, flavonoid metabolism, and anthocyanin metabolism are critical to the formation of macadamia inflorescence coloration. The phenylpropanoid biosynthesis pathway serves as a foundation for the synthesis of numerous secondary metabolites, exerting a direct influence on the production of anthocyanins and related compounds (Jiang et al., 2022). These secondary metabolites not only contribute to the plant’s defense mechanisms but also play a crucial role in attracting pollinators. Flavonoid metabolism is inextricably linked to the synthesis of anthocyanins. The regulation of specific enzyme expression within this pathway influences the intensity and variation of floral coloration (Katsumoto et al., 2007). For example, flavonoids not only participate in the biosynthesis of pigments but also impact the stability of anthocyanins, thereby affecting their distribution within petals and the visual effects that they produce (Tanaka et al., 2008; Qiu et al., 2023). In addition, the biosynthetic pathways of flavonoids, phenylpropanoid, and anthocyanins can form interrelated metabolic networks thereby dynamically regulating color phenotypes. The phenylpropanoid pathway serves as the basal pathway, producing precursors such as cinnamic acid and coumaroyl coenzyme A, which are required for downstream flavonoid synthesis (Vogt, 2010). In FC1, the dihydroflavonol reductase (DFR) and anthocyanin synthase (ANS) genes synthesize large amounts of anthocyanins, leading to anthocyanin accumulation. In contrast, FC2 exhibits higher abundance of flavonols (e.g., kaempferol derivatives) that compete with anthocyanin biosynthesis for shared substrates (e.g., dihydrokaempferol), thereby suppressing color intensity (Jiang et al., 2022). This substrate competition is also regulated by MYB transcription factors that selectively activate structural genes in specific pathways (Zhou et al., 2020). For example, MYB12 in Arabidopsis enhances flavonol synthesis while inhibiting anthocyanin accumulation (Stracke et al., 2007), a mechanism that may be preserved in macadamia nuts as well. Such interactions between pathways highlight the dynamic balance between pigmented and non-pigmented metabolites in determining floral phenotypes.

It is important to note that, despite not being a direct pigment, phenolic acid can have a significant effect on colour stability and shade through co-pigmentation and antioxidant effects (Liu et al., 2018). These findings are consistent with those of the present study, which demonstrates that phenolic acids can act as co-pigments by forming complexes with anthocyanins, thereby enhancing UV absorption and colour vibrancy (Zhao and Tao, 2015). Furthermore, phenolic acids have been shown to scavenge reactive oxygen species, thereby protecting pigments from degradation and prolonging colour retention (Jiang et al., 2022). Consequently, the interaction between phenolic acids and flavonoids underscores their collaborative role in macadamia inflorescence colouration. Ultimately, the metabolism of anthocyanins determines the phenotype of floral color. The regulatory mechanisms are characterized by an intricate interplay of multiple transcription factors and signaling pathways (Zhou et al., 2020; Chaves–Silva et al., 2018). The coordinated activity of these three pathways plays a significant role in the development and evolution of macadamia floral coloration.

In this study, traditional phenotypic and metabolomic methods were employed to explore the molecular mechanisms underlying inflorescence colour variation in Macadamia. These methods enabled the identification of key metabolites and metabolic pathways associated with different flower colours, providing a fundamental understanding of the colour variation process (**Figure 9**). However, the limitations of the approach employed are acknowledged. The categorization of flower colors was predominantly informed by phenotypic observations and PCA clustering of metabolite data, which may not have attained the objectivity that more advanced color measurement techniques can offer (**Figure 5A**). The employment of techniques such as the use of a colorimeter or PS RGB parameters has been demonstrated to facilitate a more objective and accurate quantification of flower colours. For instance, a colorimeter has been shown to enable the precise measurement of the reflectance of different wavelengths of light from the inflorescences, thereby providing numerical values that directly represent colour characteristics. Furthermore, PS RGB parameters have been demonstrated to facilitate the analysis of digital images of the inflorescences, enabling a more detailed and standardized color analysis (Wang et al., 2018; Zhou et al., 2022). The aim of this study was to establish a basic framework for understanding colour variation in macadamia inflorescences, laying the foundation for subsequent research. In order to advance this area of research, future studies intend to employ more objective colour measurement techniques, such as using colourimeter data to precisely define colour groups, followed by in-depth metabolomics analyses of each group. This is expected to improve the accuracy of chromatic classification and strengthen the correlation between colour phenotypes and specific metabolite profiles. This study offers valuable insights into the genetic and molecular basis of floral color variation, providing a foundation for further research into the mechanisms underlying these processes. Furthermore, an enhanced comprehension of these metabolic pathways may facilitate the development of novel strategies for the improvement of macadamia cultivation traits and the enhancement of its economic value.

## Data Availability

The original contributions presented in the study are included in the article/**Supplementary Material**. Further inquiries can be directed to the corresponding author/s.
