# Supplementary material for: Unravel the molecular basis underlying inflorescence color variation in Macadamia based on widely targeted metabolomics
Source: Front Plant Sci. 2025 Mar 25;16:1533187. doi: 10.3389/fpls.2025.1533187 (PMC11975671; doi:10.3389/fpls.2025.1533187)
Supplement: Supplementary file 1 [file DataSheet1.zip › Supplementary Files.docx]

**SUPPLEMEANTAL FILES**

**Unravel the molecular basis underlying inflorescence color variation in *Macadamia* based on widely targeted metabolomics**

**Lidan Gong^1^, Haiqing Zhang^1^, Jing Ma^1^, Zhiqiang Li^1^, Tingyu Li^1^, Chao Wu^1^, Yang Li^1^, Liang Tao^1*^**

^1^Yunnan Institute of Tropical Crops, Jinghong 666100, Yunnan, China

*Correspondence: Liang Tao (basanyeyu@vipsina.com)

**SUPPLEMENTAL FIGURES**

**
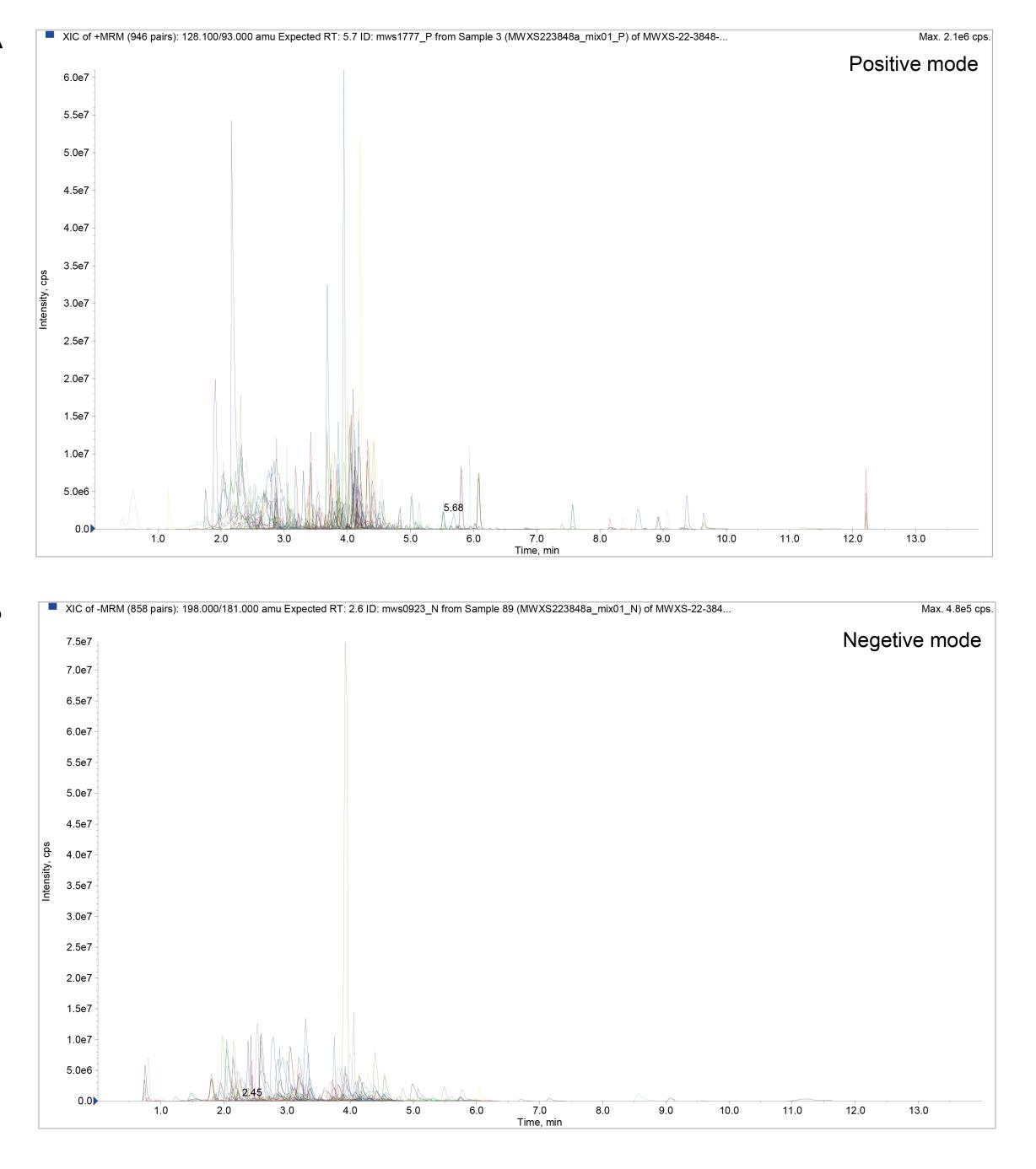
**

**Figure S1.** Qualitative and quantitative analysis of metabolites. MRM Metabolite Detection Multi-Peak Chromatogram (Ion Flow Spectrogram for Multi-Substance Extraction, XIC), the horizontal coordinate is the retention time (Rt) of the metabolite detection, and the vertical coordinate is the intensity of the ion flow of the ion detection (the intensity is in cps, count per second). Each different colored peak represents one metabolite detected. **(A)** positive ion mode and **(B)** negative ion mode.

**
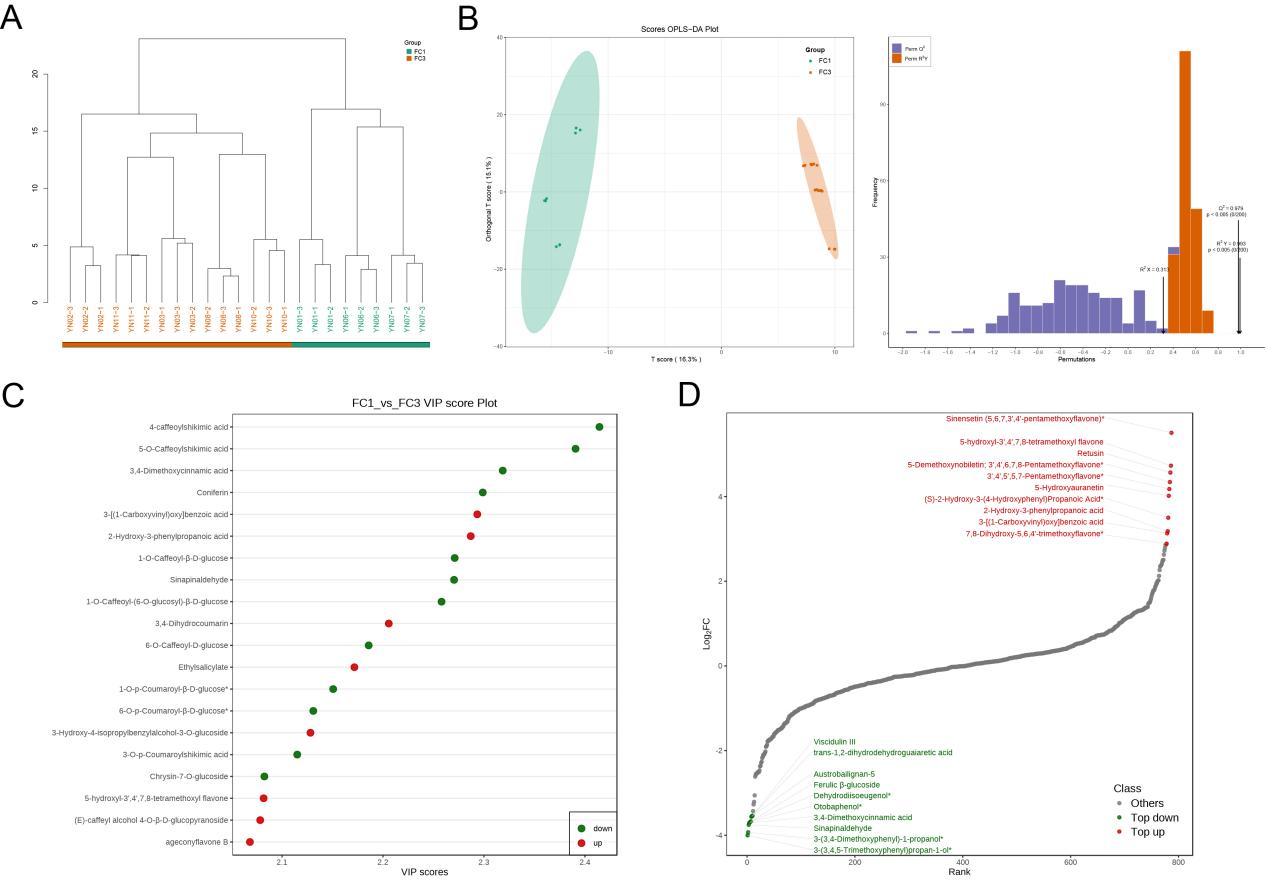
**

**Figure S2.** Screening and characterization of differential metabolites in FC1 vs FC3. (**A**) Hierarchical clustering plot of samples from the FC1 and FC3 groups. (**B**) OPLS-DA diagrams for FC1 and FC3 groups. Horizontal coordinates denote predicted principal components and vertical coordinates denote orthogonal principal components, which indicate inter- and intra-group disparities, respectively. The right-hand panel shows the validation of the OPLS-DA model. The horizontal coordinates represent the model R^2^Y, Q^2^ values, and the vertical coordinates are the frequency of the model classification effects appearing in 200 random permutation experiments. (**C**) Map of the top 20 differential metabolites with the largest VIP values in FC1 vs FC3. (**D**) Plot of the dynamics of the top 10 up- and down-regulated metabolite contents with the largest fold change in FC1 vs FC3.


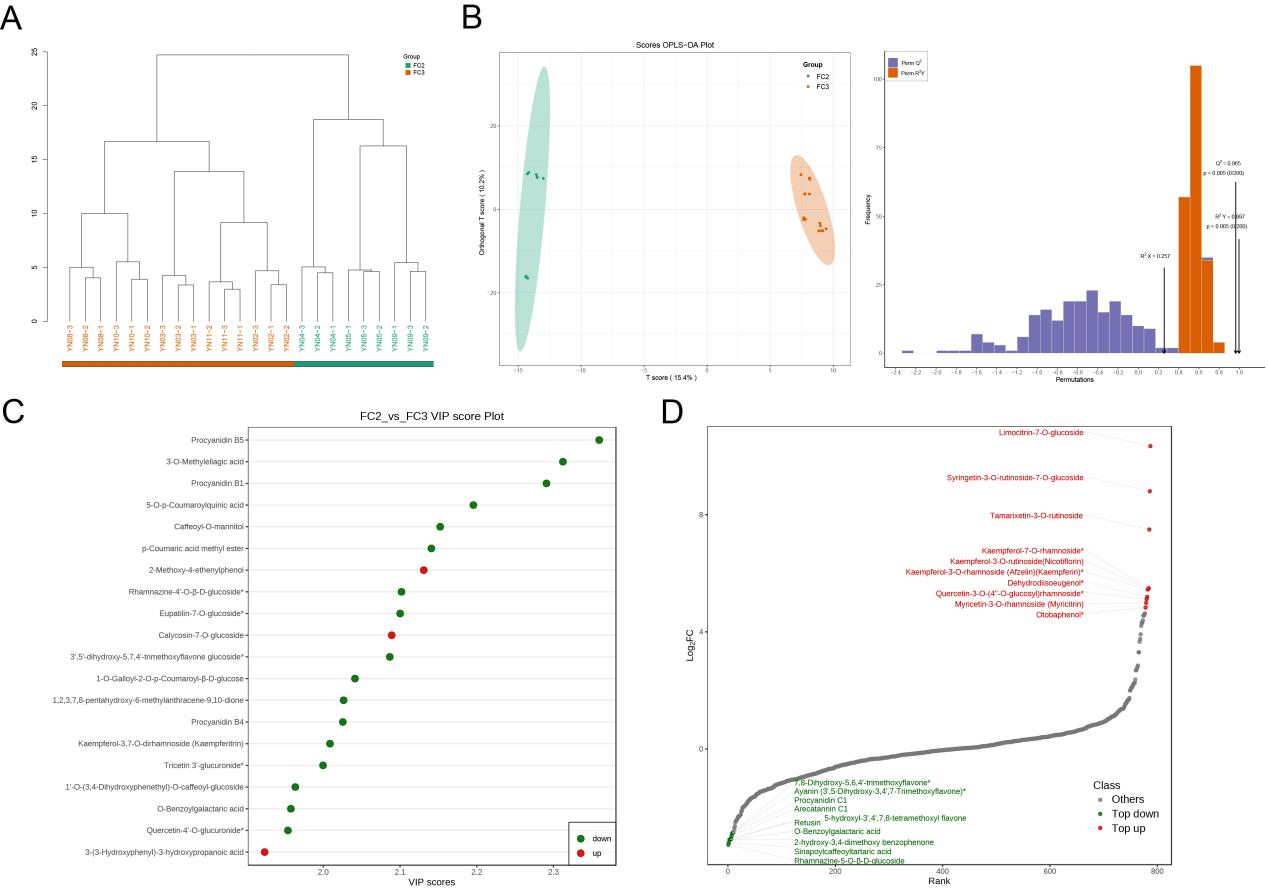


**Figure S3.** Screening and characterization of differential metabolites in FC2 vs FC3. (**A**) Hierarchical clustering plot of samples from the FC2 and FC3 groups. (**B**) OPLS-DA diagrams for FC2 and FC3 groups. Horizontal coordinates denote predicted principal components and vertical coordinates denote orthogonal principal components, which indicate inter- and intra-group disparities, respectively. The right-hand panel shows the validation of the OPLS-DA model. The horizontal coordinates represent the model R^2^Y, Q^2^ values, and the vertical coordinates are the frequency of the model classification effects appearing in 200 random permutation experiments. (**C**) Map of the top 20 differential metabolites with the largest VIP values in FC2 vs FC3. (**D**) Plot of the dynamics of the top 10 up- and down-regulated metabolite contents with the largest fold change in FC2 vs FC3.

**
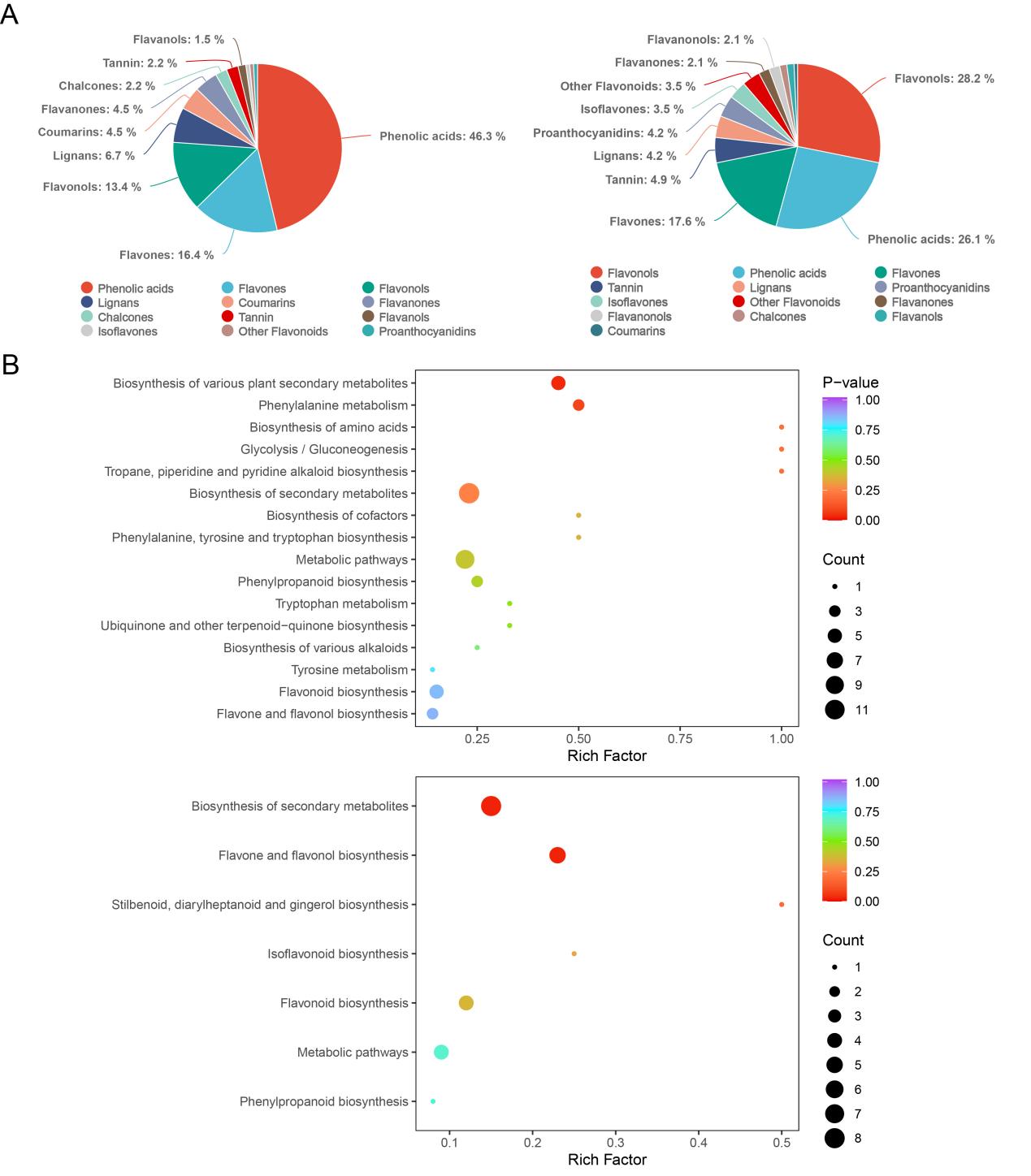
**

**Figure S4.** Identification and enrichment analysis of differential metabolites. (**A**) Pie charts of secondary categorization of differential metabolites in FC1 vs. FC3 (left panel) and FC2 vs. FC3 (right panel). (**B**) Bubble plots of KEGG enrichment analysis of differential metabolites in FC1 vs. FC3 (upper panel) and FC2 vs. FC3 (lower panel). The horizontal coordinate indicates the corresponding Rich Factor for each pathway, the vertical coordinate is the pathway name, the color of the dot reflects the P-value, and the size of the dot represents the number of differential metabolites.

**
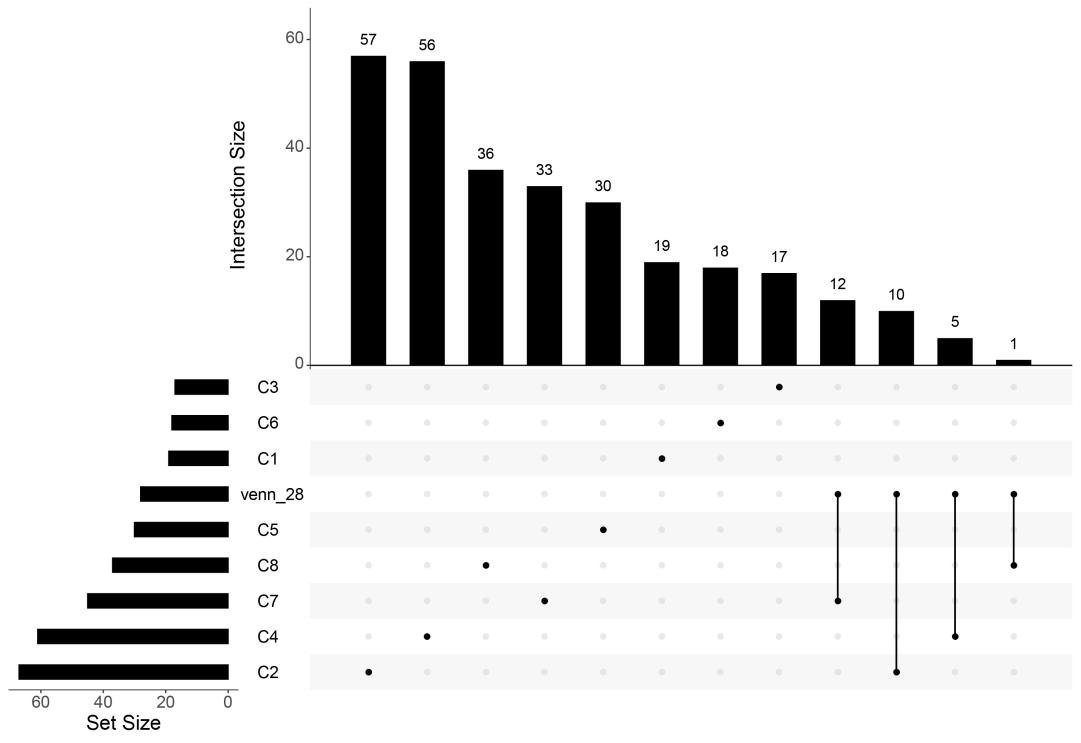
**

**Figure S5.** Upset plot of differential metabolites. The figure presents the 28 metabolites that were co-identified in the three comparison groups in relation to the eight metabolites with different expression trends in the K-Means analysis.


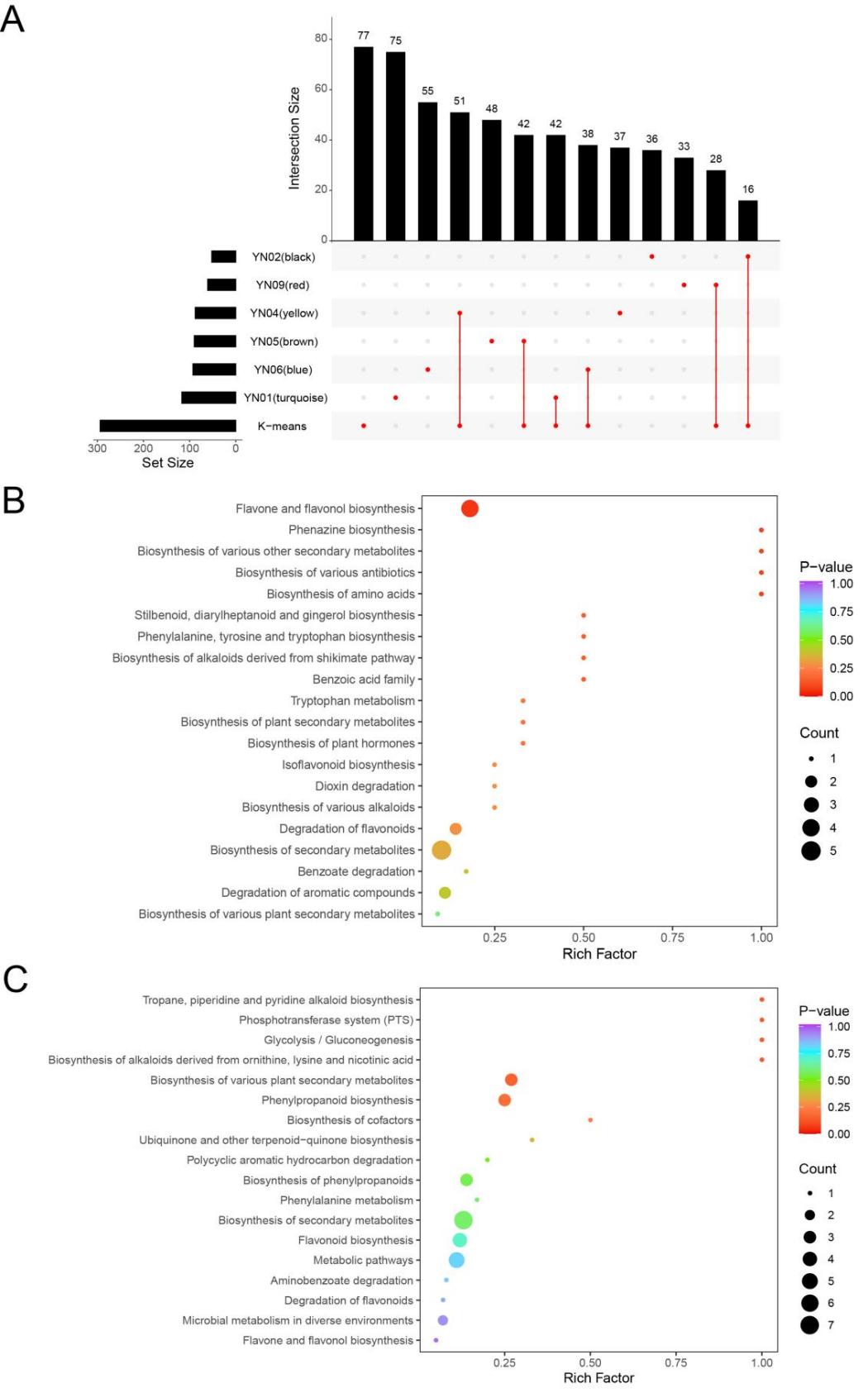


**Figure S6.** WGCNA analysis combined with K-Means analysis to identify key metabolites of inflorescence color. (**A**) Upset plot of key metabolites. Intersection of metabolites from the top 6 modules with the highest correlation in the WGCNA analysis with metabolites from the K-Means analysis. Plot of KEGG enrichment analysis of differential metabolites belonging to modules in FC2 (**B**) and FC1 (**C**) from WGCNA analysis.
