# Supplementary figures and images for: Unravel the molecular basis underlying inflorescence color variation in Macadamia based on widely targeted metabolomics
Source: Front Plant Sci. 2025 Mar 25;16:1533187. doi: 10.3389/fpls.2025.1533187 (PMC11975671; doi:10.3389/fpls.2025.1533187)

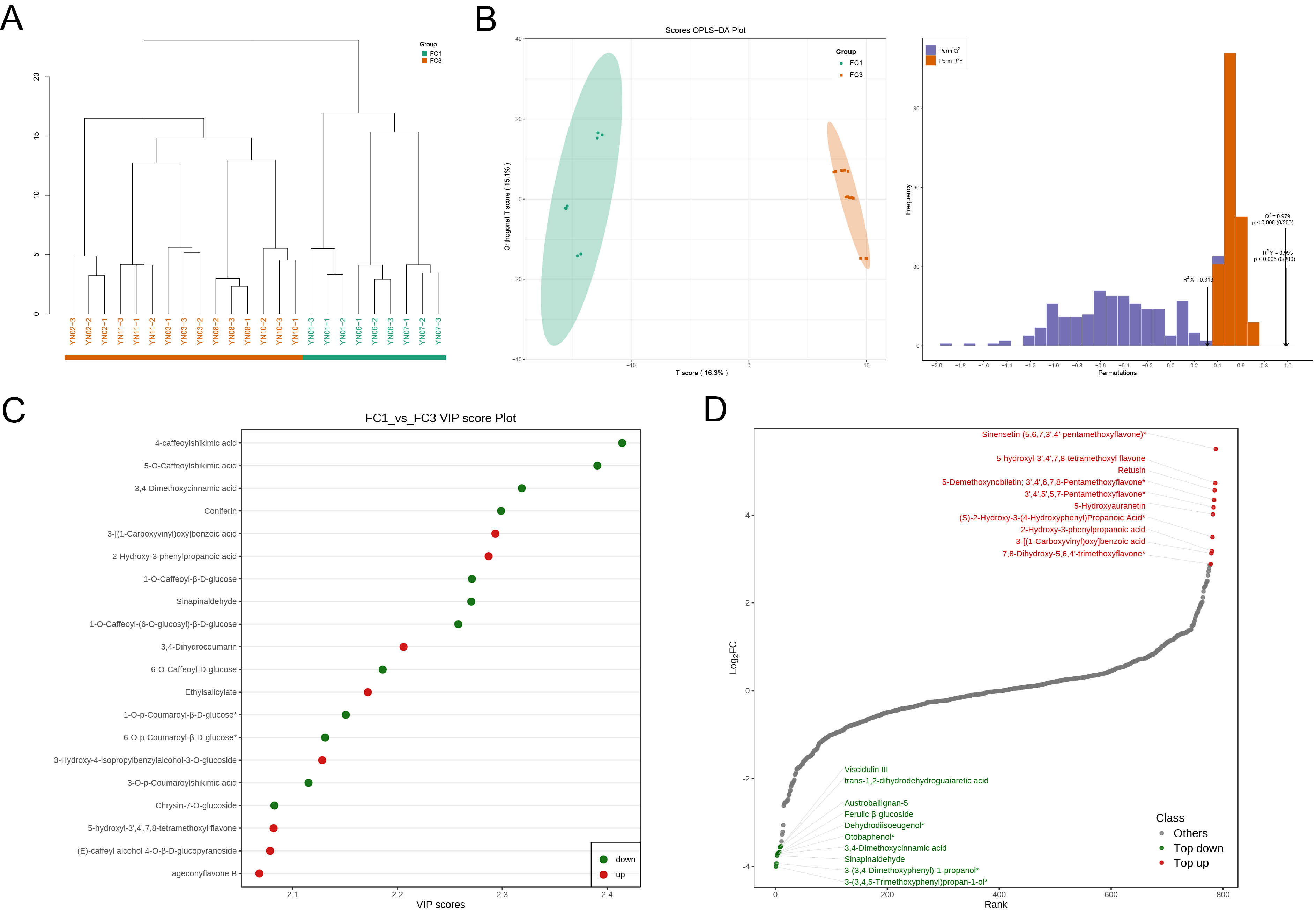

Supplement: Supplementary file 1 [file DataSheet1.zip › Supplementary Figure 2.jpg]

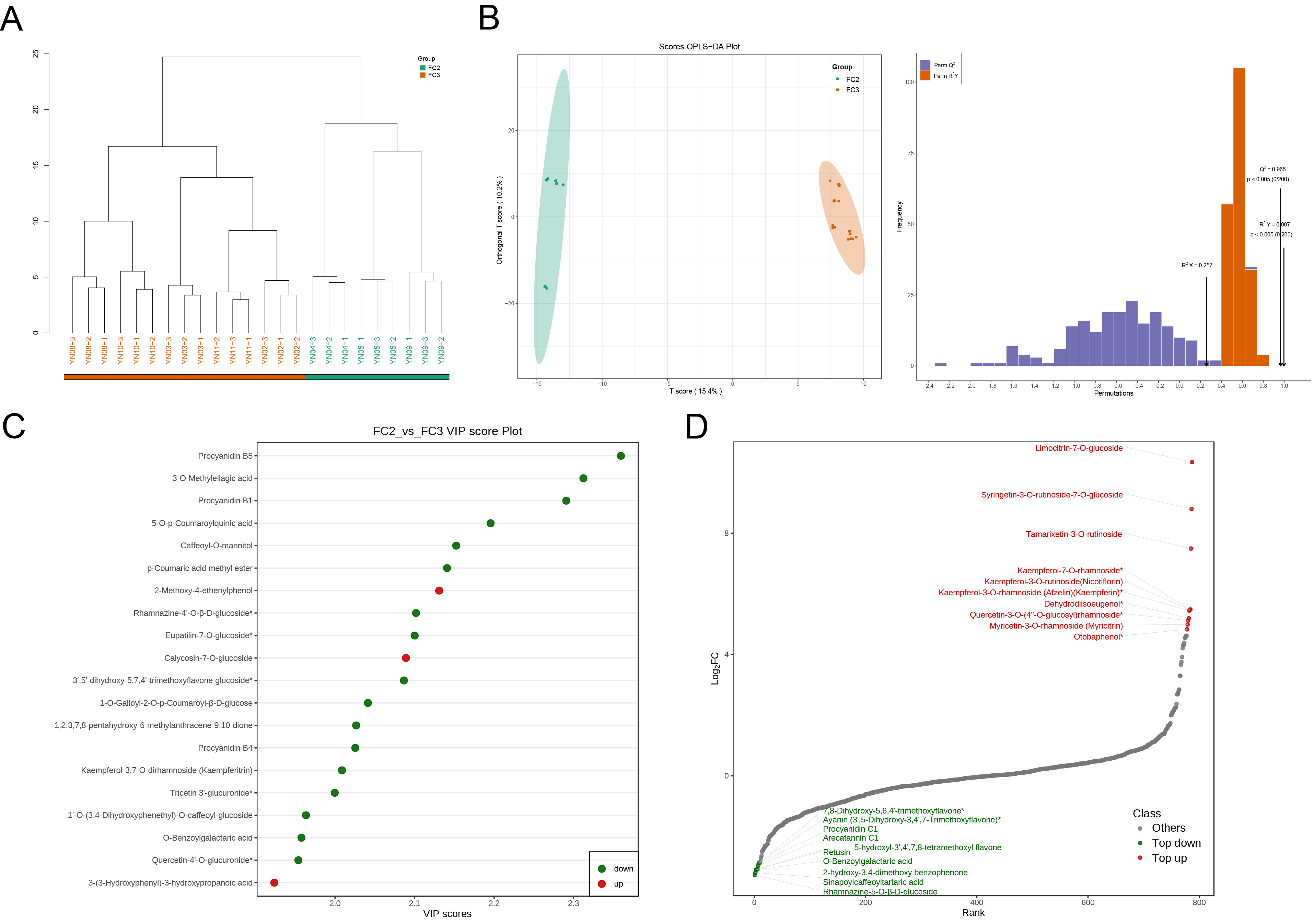

Supplement: Supplementary file 1 [file DataSheet1.zip › Supplementary Figure 3.jpg]

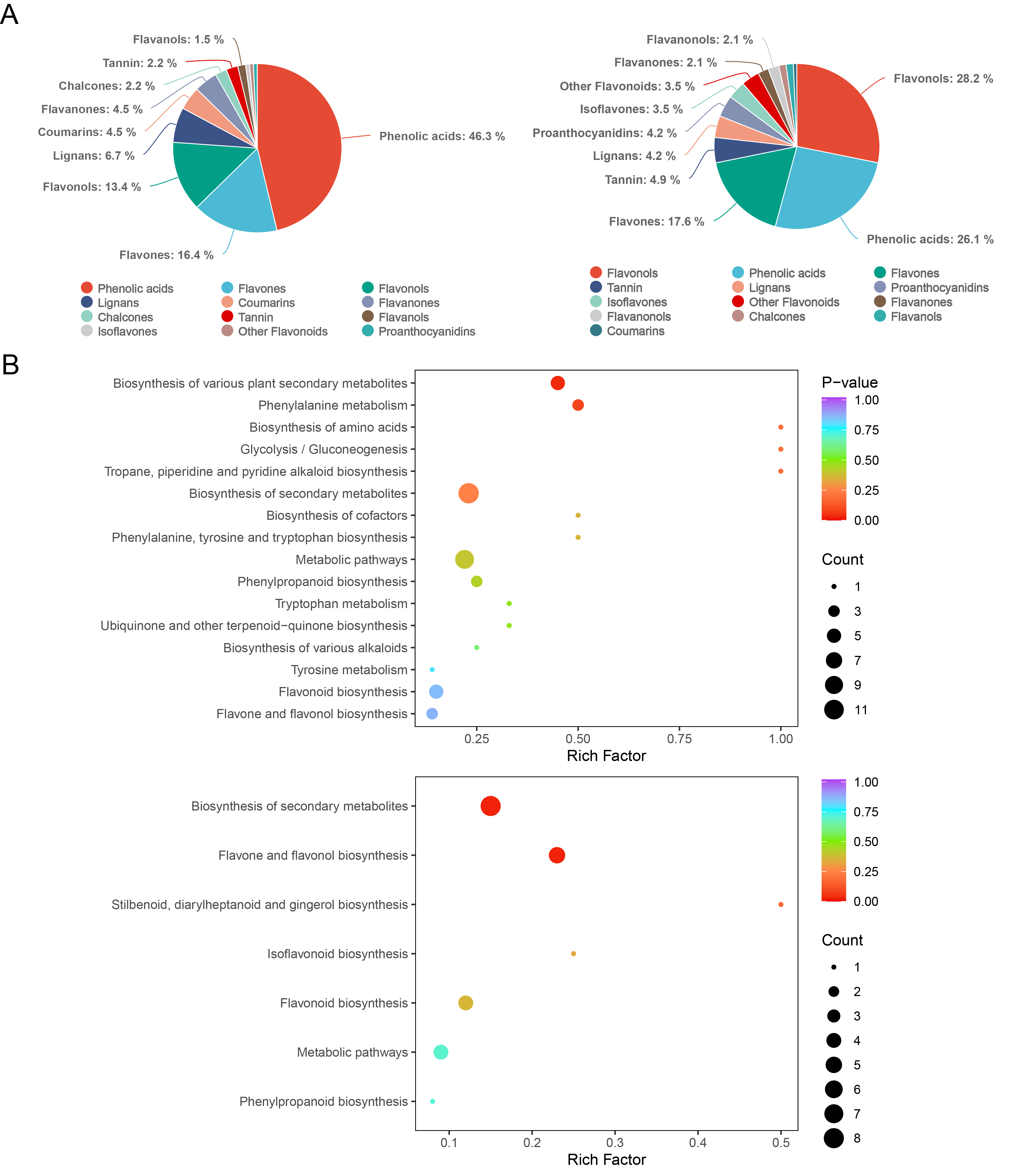

Supplement: Supplementary file 1 [file DataSheet1.zip › Supplementary Figure 4.jpg]

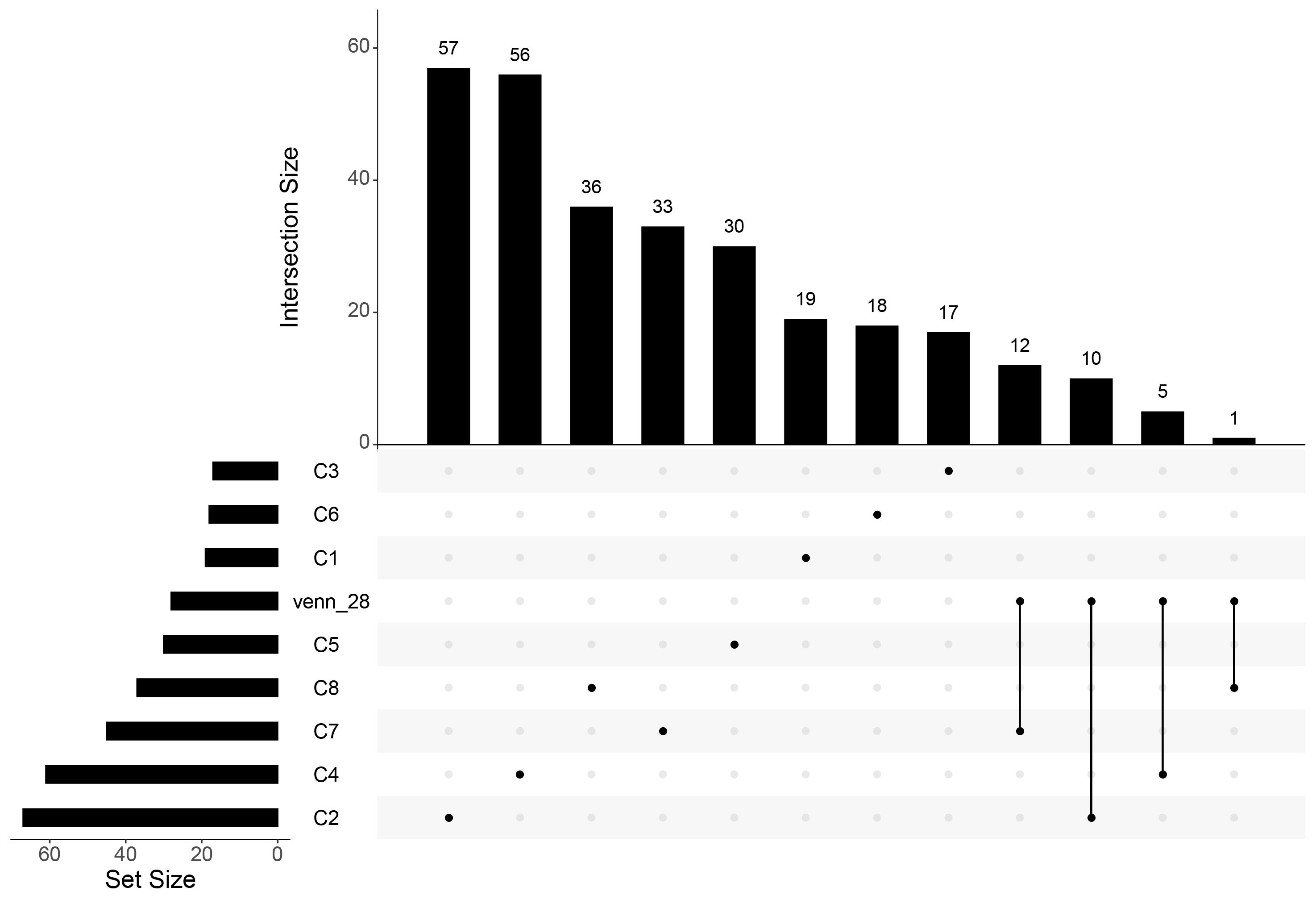

Supplement: Supplementary file 1 [file DataSheet1.zip › Supplementary Figure 5.jpg]

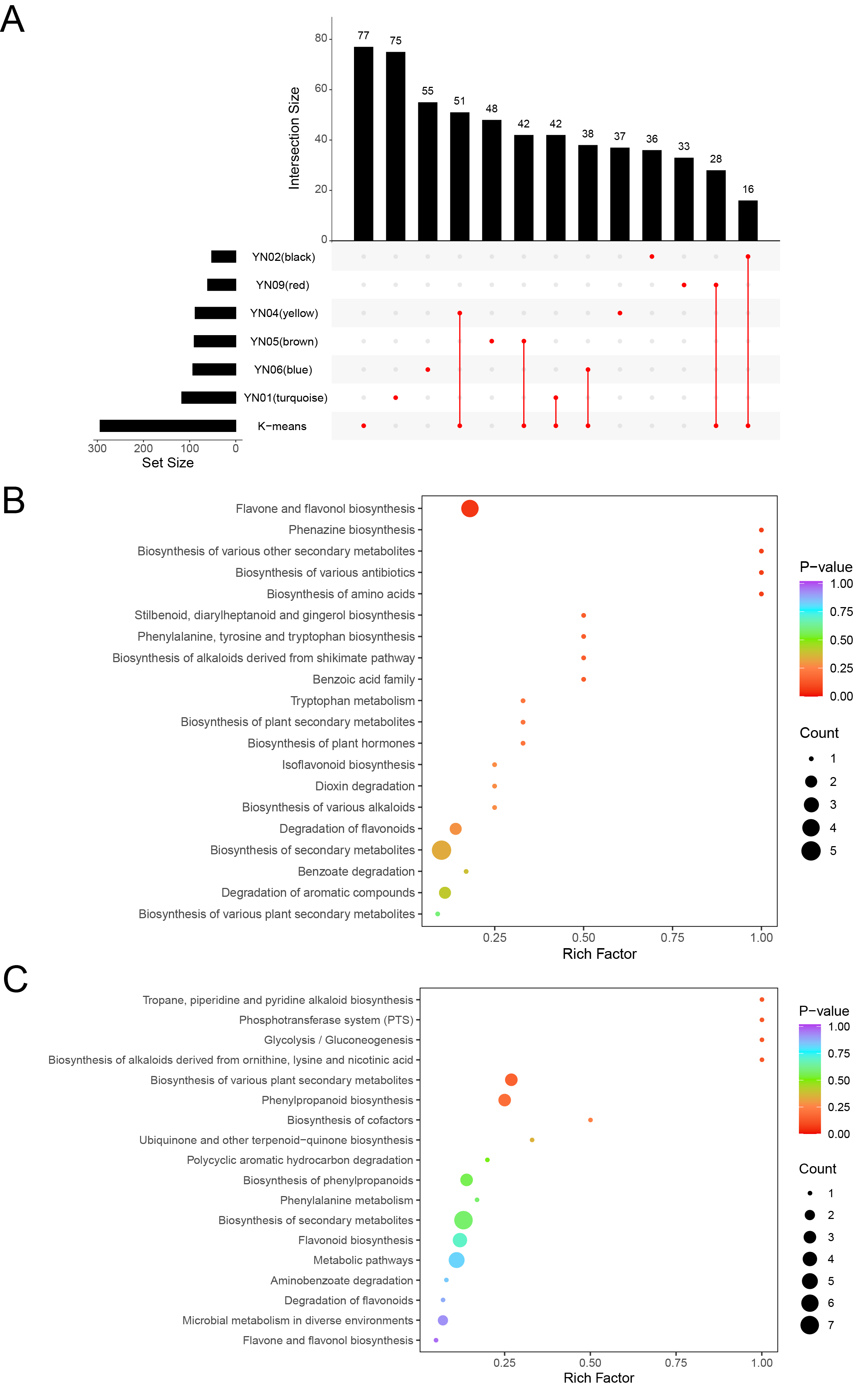

Supplement: Supplementary file 1 [file DataSheet1.zip › Supplementary Figure 6.jpg]

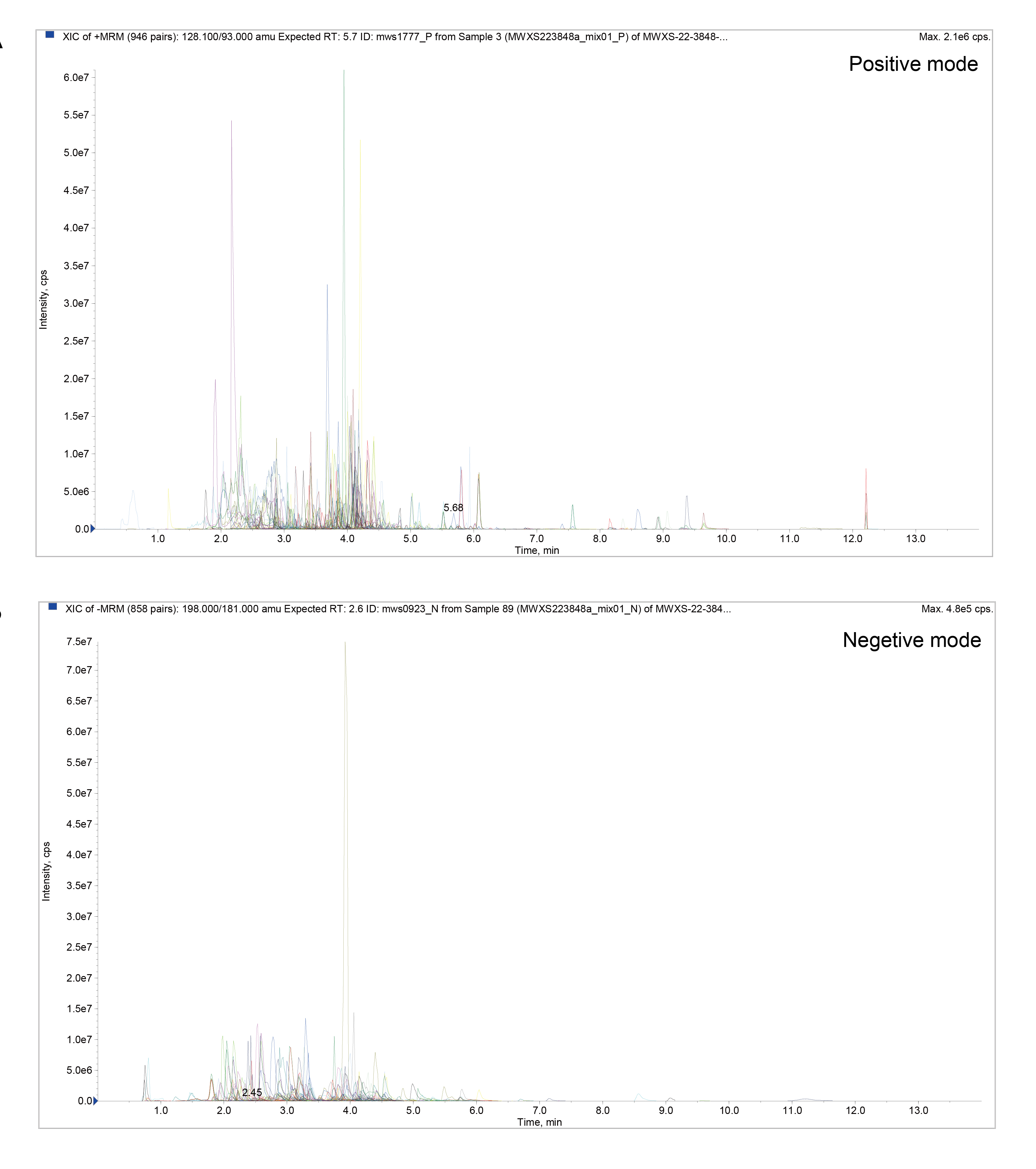

Supplement: Supplementary file 1 [file DataSheet1.zip › Supplementary Figure 1.jpg]
